# Integrating epidemiological, regulatory and social perspectives to improve bovine leukemia virus control

**DOI:** 10.1007/s11259-026-11563-z

**Published:** 2026-10-31

**Authors:** Camille Demassieux, Nicolas Rose, Guillaume Lhermie, Alexandre Hobeika, Stephen Valas

**Affiliations:** 1 Ploufragan-Plouzané-Niort Laboratories, French Agency for Food, Environmental and Occupational Health & Safety (ANSES), Maisons-Alfort, France; 2 Faculty of Veterinary Medicine, University of Calgary, Calgary, Canada; 3 MOISA Research Unit, French Agricultural Research Centre for International Development (CIRAD), Montpellier, France

**Keywords:** Bovine leukemia virus (BLV), Enzootic bovine leukosis (EBL), Infectious disease control strategy, Veterinary public health, Epidemiological surveillance, Social implication of disease management

## Abstract

Enzootic bovine leukosis (EBL), caused by the bovine leukemia virus (BLV), is a widespread bovine disease responsible for chronic economic losses in the cattle industry. Prevalence has risen substantially in countries without control measures, whereas state‑led programs have generated a more heterogeneous epidemiological landscape across Europe. This review examines key aspects of BLV impact and control in both global and European contexts. Using publicly available datasets from the World Organisation for Animal Health, it summarizes current BLV epidemiology and the outcomes of current control strategies. It also reviews emerging control options for high‑prevalence settings and challenges in economic assessment. Finally, it uses established findings from social science studies applied to veterinary public health to analyze issues linked to BLV control programs. The impact of BLV was greater than previously assumed, particularly in countries with high prevalence. Regions with low prevalence should not be overlooked, given the increasing occurrence of emerging diseases and the expansion of insect vectors associated with climate change. The economic cost of inaction may be increasing, as recent studies have suggested a potential but still controversial zoonotic capacity that may already affect the reputation of animal products in endemic areas. Beyond the farming sector, BLV-related social costs could include political instability stemming from social burdens generated by veterinary public health programs. Altogether, these observations highlight the need for a cross-sectoral approach to animal disease management in veterinary public health, integrating emerging concerns related to human health, environmental degradation, and political instability in a One-Health perspective.

**Supplementary Information:**

The online version contains supplementary material available at https://doi.org/10.1007/s11259-026-11563-z.

## Background

Enzootic bovine leukosis (EBL) is one of the most widely distributed bovine diseases in the world, especially among intensive farming systems, causing chronic economic losses in the cattle industry (Thurmond 1986; Ott et al. 2003; EFSA AHAW Panel 2015). EBL is a contagious bovine neoplastic disease caused by the oncogenic bovine leukemia virus (BLV) (WOAH 2018).

BLV belongs to the *Deltaretrovirus* genus of the *Retroviridae* family (International Committee on Taxonomy of Viruses 2025). It establishes a lifelong but mostly asymptomatic infection through integration of its genome into host chromosomes. BLV pathogenesis is driven by early viral replication followed by immune control, with long-term outcomes ranging from latent infection to persistent lymphocytosis and, in 5–10% of cases, the development of fatal lymphosarcoma. The tumor incubation period is influenced by viral genotypes (Mori et al. 2019), age of infection, specific provirus integration sites (Maezawa and Inokuma 2020) and the host’s genetic background (Akbarin et al. 2025). Although its clinical impact is moderate, the economic costs of BLV have been considered increasingly salient and are detailed in several recent reviews, highlighting its influence on multiple aspects of bovine management, from production losses to decreased animal fertility and higher rates of co-morbidities in infected cows (Bartlett et al. 2014, 2020; Kuczewski et al. 2021b; Lv et al. 2024; Bartlett 2025). As a result, the substantial increase in BLV prevalence in countries where no control measures have been implemented underscores the need for its control (Bartlett 2025). Additionally, recent studies have suggested a potential foodborne transmission risk of BLV to humans through fresh unpasteurized milk and raw beef, potentially causing breast cancer, and corroborating public apprehension (Buehring et al. 2019; Buehring and Sans 2020; Gao et al. 2020; Mendoza et al. 2023; Blanco et al. 2025; Moran et al. 2025). Although no scientific consensus has yet been established regarding this zoonotic potential, it has nonetheless intensified concerns related to BLV control strategies among citizens and scientists (Gyles 2016; Bartlett 2025).

In North America and Asia, no control program has been implemented against the spread of BLV, resulting in a sustained increase in prevalence among both beef and dairy cattle. Dairy herds were found to be the most affected, with herd-level prevalence ranging from 83.9% in the United States (US), to 86.7% in Canada, and 86.8% in South Korea (Suh GukHyun et al. 2003; Scott et al. 2006; Centers for Epidemiology and Animal Health 2008). In Europe, BLV control measures have been implemented for many decades. Health regulations cover both live animals and products of animal origin (European Parliament and Council of European Union 2016). Although state-implemented control measures targeting BLV became optional in 2018 (European Commission 2018), EBL remains the disease with the highest number of control programs in the European Union (EU) (Costa et al. 2020; Hodnik et al. 2021). As of 2025, 21 EU Member States are officially recognized as free from BLV according to EU regulations, allowing facilitated cattle trade between these countries (European Commission 2026a). Only 5 out of the 27 EU Member States and 2 EU outermost regions (ORs) (European Parliament 2025), (Madeira Island/Portugal and Reunion Island/France) are not yet free from the disease (European Commission 2026a).

On a technical level, long-established BLV control plans rely on extensive knowledge accumulated within the EU since the 1960s. In addition, reviews have proposed complementary technical approaches and innovative strategies for controlling high-prevalence herds, which are adapted to North American settings (Bartlett et al. 2014, 2020; Kuczewski et al. 2021b). However, these innovations have emerged in a context where several phenomena linked with climate change—such as heat stress, shifts in vector populations, and the emergence of new infectious diseases—are creating new challenges that need to be integrated into all aspects of veterinary public health, including the control of BLV (Hempel et al. 2019; Carvajal et al. 2021; de Souza and Weaver 2024; Ilevičius 2025). Additionally, at a societal level, the costs arising from mistrust of meat products, distrust of the competent authorities, or the difficulty in securing livestock farmers’ support for control programs, are aspects that are rarely discussed. To our knowledge, no previous research has examined the broader societal burden, governance challenges, and issues of stakeholder acceptance associated with BLV control policies, although some of these policies have generated significant contestation.This review aims to inform decision-makers by identifying which approaches and parameters should be considered when designing BLV control strategies. It first provides a spatial and temporal overview to clarify the global and European epidemiological context for BLV, alongside an analysis of current knowledge regarding its transmission pathways. To this aim, we used the literature on BLV and its epidemiology alongside publicly available EU regulations, and we analyzed datasets from the World Animal Health Information System (WAHIS) of the World Organisation for Animal Health (WOAH) using a geographic information system and statistical treatment (QGIS.org 2024; Wickham et al. 2024; R Core Team 2024). We considered political Europe (European Union) as of August 2023, together with partner countries of continental Europe like Great Britain, Iceland, Switzerland, and Norway, as well as non-continental and overseas territories (outermost regions) of EU countries (European Commission, Directorate General for Communication 2023; European Parliament 2025). We then focus on the context in which BLV control programs are developed, by adopting a systemic approach for the assessment of BLV impact and control, broadening the veterinary public health perspective. This includes emerging interests for controlling BLV and opportunities provided by innovative control methods. It also integrates social analysis, deemed necessary to address the governance challenges currently encountered in veterinary public health, notably seeking to describe the cost of the social burden of both BLV and its control strategies.

## Global and European approaches to BLV epidemiology

### BLV transmission pathway

The only recognized reservoir of BLV infection is live cattle. Indeed, the only known infectious material for BLV consists of biological fluids containing infected cells, as cell-free virions are generally not detected in vivo (Evermann et al. 1986). Primary infection occurs after contact of the organism with blood (or any biological secretions derived from blood containing infected B lymphocytes), through cutaneal effraction (whether or not iatrogenic), oral pathways, or direct contact of mucosal surfaces like the mouth and nose (WOAH 2018).

Variable findings about the likelihood of BLV transmission are explained by greatly varying infection rates among cattle, i.e., the proviral load (PVL) in the peripheral blood of infected cattle. PVL variation in peripheral blood is influenced by the host’s genetic polymorphisms at the major histocompatibility complex (Fujii et al. 2026). Once settled in the host organism, PVL is stable for prolonged periods of time in most animals, with minor fluctuations (Gutiérrez et al. 2014; Hutchinson et al. 2020, 2021), before eventually rising as the disease progresses, leading to the onset of EBL (Lo et al. 2020). PVL is correlated with the secretion of infected cells in various fluids (milk, nasal mucus, and saliva) (Yuan et al. 2015; Watanuki et al. 2019). The presence of provirus in colostrum and milk is positively correlated with PVL in blood (Gutiérrez et al. 2014; Watanuki et al. 2019; Nakatsuchi et al. 2022), and milk-borne transmission from dams with high blood PVLs is significantly increased (Gutiérrez et al. 2014; Mekata et al. 2021). More broadly, several studies have demonstrated that the likelihood of BLV transmission is strongly associated with the PVL of the source animal. In particular, cattle with low PVLs appear unlikely to act as significant sources of infection under normal farming conditions, whereas animals with high PVLs are more likely to contribute to both horizontal and vertical transmission (Mekata et al. 2015, 2018; Sajiki et al. 2017). Several studies investigating BLV transmission under experimental or field conditions overlooked the PVL of source animals, limiting the reliability and reproducibility of their findings (Roberts et al. 1981; Panei et al. 2019; Benitez et al. 2019; John et al. 2020). Additionally, body fluids such as semen, nasal secretions, saliva, urine, and feces typically contain few lymphocytes—unless inflammation is present—further complicating interpretation (Hopkins and DiGiacomo 1997). Although this may have led to an underestimation of the relative importance of certain transmission pathways, the overall patterns in the relative contribution of most transmission routes are established. Bloodborne transmission remains the primary route of BLV spread, with numerous routine husbandry procedures capable of transferring infection through minimal volumes of contaminated blood. Close direct contact—particularly muzzle-to-muzzle interactions—significantly increases within-herd transmission and amplifies the risk associated with introducing animals of unknown infection status. Network analysis clearly demonstrated correlation between animal movements and the risk of BLV introduction (Notsu et al. 2020). Vertical transmission can occur in utero, at parturition, or through colostrum and milk, with colostrum-mediated exposure and post-natal contact representing the most influential pathways in determining infection risk among calves.

BLV can be detected in biting flies, and successful transmission under experimental conditions has been achieved in cattle following inoculation of homogenized mouthparts of biting flies having fed on blood from a BLV-positive cow (Bech-Nielsen et al. 1978; Buxton et al. 1985; Foil et al. 1988; Ooshiro et al. 2013; Panei et al. 2019; Shimizu et al. 2024). Some experiments conducted in a controlled environment have led to the identification of insect species capable of transmitting BLV through a natural feeding route, but most transmission assays conducted in natural grazing conditions failed (Table 1). However, transmission by biting insects has been suspected in observational surveys conducted on commercial facilities (Hopkins and DiGiacomo 1997; Kuczewski et al. 2021b) and theorized again by cross-sectional risk factor studies (Kobayashi et al. 2010). Indeed, field studies in the US, France, and Japan have reported that the seroconversion rate of BLV in beef and dairy cattle herds increased during the summer season and was associated with the density and activity of hematophagous insects (Bech-Nielsen et al. 1978; Manet et al. 1989; Kobayashi et al. 2014; Kohara et al. 2018), while the implementation of fly control measures led to a decrease in prevalence (Onuma et al. 1980; Manet et al. 1989; Erskine et al. 2012; Ooshiro et al. 2013; Kohara et al. 2018). However, other prospective studies yielded the opposite results (Wilesmith et al. 1980; Thurmond et al. 1983; Sprecher et al. 1991; Hopkins and DiGiacomo 1997). Confounding factors were suspected to underlie the difficulty in quantifying the relative contribution of vector-borne transmission to the epidemiology of BLV, leading its potential role to be viewed as controversial (Kuczewski et al. 2021b). As a result, the role of insects as carriers of BLV between bovines remains heterogeneously characterized. To our knowledge, no existing survey has examined the relationship between these heterogeneous findings and local ecological or climatic factors. This discrepancy raises questions about whether mechanical transmission by biting insects plays a significant role in BLV transmission under different environmental conditions worldwide. Most reviews reported successful European control plans even without implementing vector control measures, implying that—at least in temperate European systems—insects alone cannot sustain endemic BLV (EFSA AHAW Panel 2015). The presence of blood-sucking arthropods on farms has nonetheless been identified as a special threat for BLV in particular geographic areas, such as in the wetlands of Poland (Pluta et al. 2026) or in the Danube Delta, where fly control may fall under strict regulations given its UNESCO World Heritage Site status (Irimia et al. 2021), and in European Outermost Regions with a tropical climate, like Reunion Island where *Stomoxys* spp. pose a particular threat due to their tendency to proliferate in cattle housing facilities (Bouyer et al. 2011). Overall, insects of the *Tabanus* and *Stomoxys* genus are, to date, considered a relevant risk factor for BLV transmission in tropical or subtropical conditions (French OR of Reunion Island, Japan’s Okinawa Island, see Table 1). Overall, current evidence suggests that arthropod-mediated BLV transmission is context-dependent (Table 1). Among the species investigated, insects of the *Tabanus* genus appear to be the most strongly supported biological candidates for mechanical transmission, while *Stomoxys* spp. are considered probable vectors in some tropical and subtropical settings (French OR of Reunion Island, Japan’s Okinawa Island). In contrast, evidence supporting the role of *Haematobia irritans* and *Culicoides* spp. remains limited. Taken together, available data suggest that biting insects may contribute to BLV transmission, at least under specific ecological conditions, but their overall importance in sustaining endemic BLV circulation remains poorly assessed and warrants further investigation.

**Table 1:** Known insect vector species in BLV transmission

Biting and sucking insect species	Common name	Presence in Europe or in European outermost regions	Results of experimental vector-borne transmission of BLV	Current level of evidence
*Haematobia irritans*	Horn Fly	Yes	Sequencing detected provirus in mouthparts (Panei et al. 2019). Transmission confirmed through experimental inoculation of crushed mouthparts (Buxton et al. 1985). Transmission experiments under controlled or natural grazing conditions did not demonstrate transmission (Buxton et al. 1985; Panei et al. 2019)	Insufficient evidence
*Culicoides* spp.	Midges	Yes	Transmission not demonstrated, but phylogenic analysis found the same substitutions in the BLV genome between cattle and a BLV-positive pool of Culicoides (Dogan et al. 2020). BLV DNA detected in in field-collected specimens (Pluta et al. 2026)	Insufficient evidence
*Stomoxys* spp.	Stable Fly	Yes	Detection of virus in mouthparts and blood meals (Freitas and Romero 1991; Shimizu et al. 2024). BLV DNA detected in in field-collected specimens (Pluta et al. 2026). Transmission confirmed through experimental inoculation of crushed mouthparts (Buxton et al. 1985). Failed transmission experiment under controlled experimental conditions (Buxton et al. 1985; Weber et al. 1988). Although scientific consensus considers their role not to be demonstrated (Baldacchino et al. 2013), *Stomoxys* flies are considered a relevant transmission pathway in tropical or subtropical conditions (French OR of Reunion Island, Japanese Okinawa Island) (Bouyer et al. 2011; Ooshiro et al. 2013)	Probable
*Tabanus* spp.	Horse Fly	Yes	Transmission confirmed through experimental inoculation of crushed mouthparts (Buxton et al. 1985). Successful transmission under controlled experimental conditions from cow to goat by a group of more than 50 horse flies (Foil et al. 1988). Tabanids are considered a relevant transmission pathway in field conditions in Japan (Kobayashi et al. 2015; Inagaki et al. 2019)	Probable

**Fig. 1:**
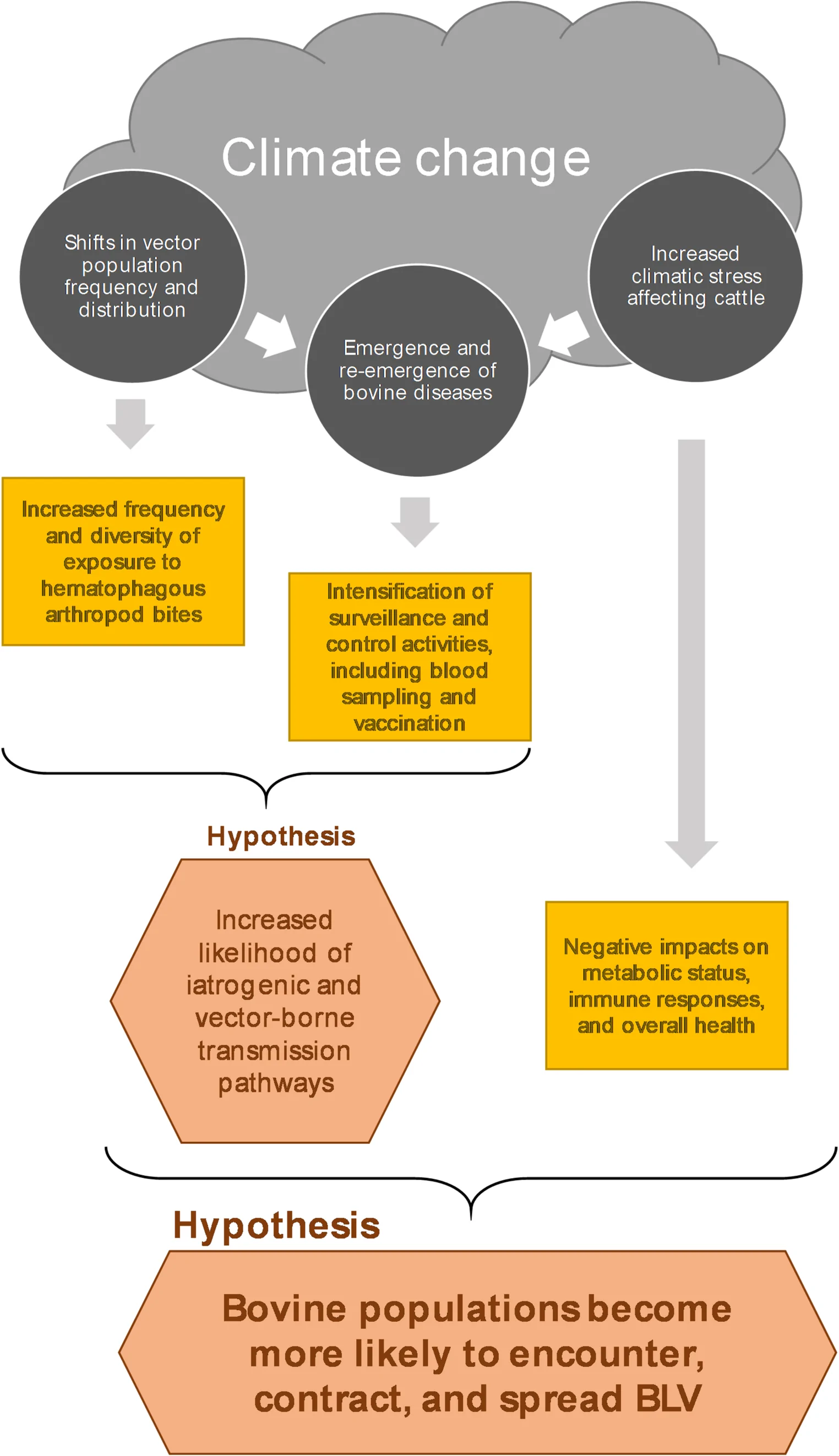
Conceptual framework illustrating potential impacts of climate change on BLV epidemiology and management challenges in Europe. Several pathways shown are hypothetical and require further investigation

Climate change may influence the future epidemiology of BLV through several mechanisms, although the available evidence remains largely indirect and these pathways should presently be regarded as hypotheses requiring further investigation (Fig. 1). Historically, temperate regions are likely to become increasingly suitable for potential BLV vectors, such as *Stomoxys* flies, leading to a redistribution of vector populations across Europe (de Souza and Weaver 2024; Li et al. 2025). More broadly, climate change is likely to promote the vector-borne expansion of bovine diseases, as illustrated by the recent geographic spread of lumpy skin disease into the EU in 2025 (ANSES 2025). In addition, heat stress resulting from climatic conditions exceeding the thermoneutral zone of ruminants may impair immune function, thereby increasing cattle’s susceptibility to infection (Collier et al. 2006; Hempel et al. 2019). If biting insects contribute substantially to BLV transmission under specific environmental conditions, these climatic changes could potentially affect BLV transmission dynamics and facilitate local virus persistence or spread, even in low-prevalence territories, resulting in a renewed BLV spread from persistent infectious clusters within Europe. However, direct evidence supporting such effects remains limited. Furthermore, the emergence or re-emergence of infectious diseases may increase the number of management programs relying on blood sampling or vaccination (WOAH 2025b). Because iatrogenic transmission of BLV can occur when biosecurity procedures are not adequately applied, a greater frequency of such interventions could theoretically increase transmission opportunities.. Nevertheless, this mechanism remains speculative and has not been demonstrated at a population level. In this context, failure to address BLV could lead to wider dissemination of the virus and a sharp increase in its impact, as observed in North American facilities where BLV may interferes with veterinary public health objectives. Taken together, these observations highlight several plausible pathways through which climate change and evolving disease-control practices might influence future BLV epidemiology. However, these scenarios remain hypothetical and warrant dedicated epidemiological studies before any firm conclusions can be drawn.

### Global epidemiology of BLV

The global epidemiological status of BLV has been heterogeneously evaluated by scientific studies, as each nation has its own scientific program and/or official monitoring investigation by local authorities. By comparison, WOAH data on the BLV status of the reporting countries offer a standardized and harmonized reporting framework for BLV cases and BLV status of WOAH members (WAHIS 2025, 2026a) and Appendix II). In WOAH reports, a case is defined as one or more infected animals, expressed as heads, regardless of the presence of clinical signs, and includes animals that died from the disease (WOAH 2025a). To analyze the epidemiological situation, the country/territory was used as the epidemiological unit, because BLV status may differ across territories belonging to the same WOAH Member. By contrast, information on control measures was analyzed at the WOAH Member level, since such measures are generally implemented uniformly at the Member scale and apply across all its associated territories. This distinction made it possible to account for territorial heterogeneity in disease occurrence while preserving coherence in the assessment of control strategies. The fragmented nature of the WOAH data for years 2021 to 2025 required the production of aggregated datasets combining the reported status of several years to design a map representing each country or territory. As these aggregated representations did not provide a picture of the global epidemiological situation at a given period, we chose to focus on the dataset for 2024, because it was the most complete and up-to-date, with full data on most countries and territories.

**Fig. 2:**
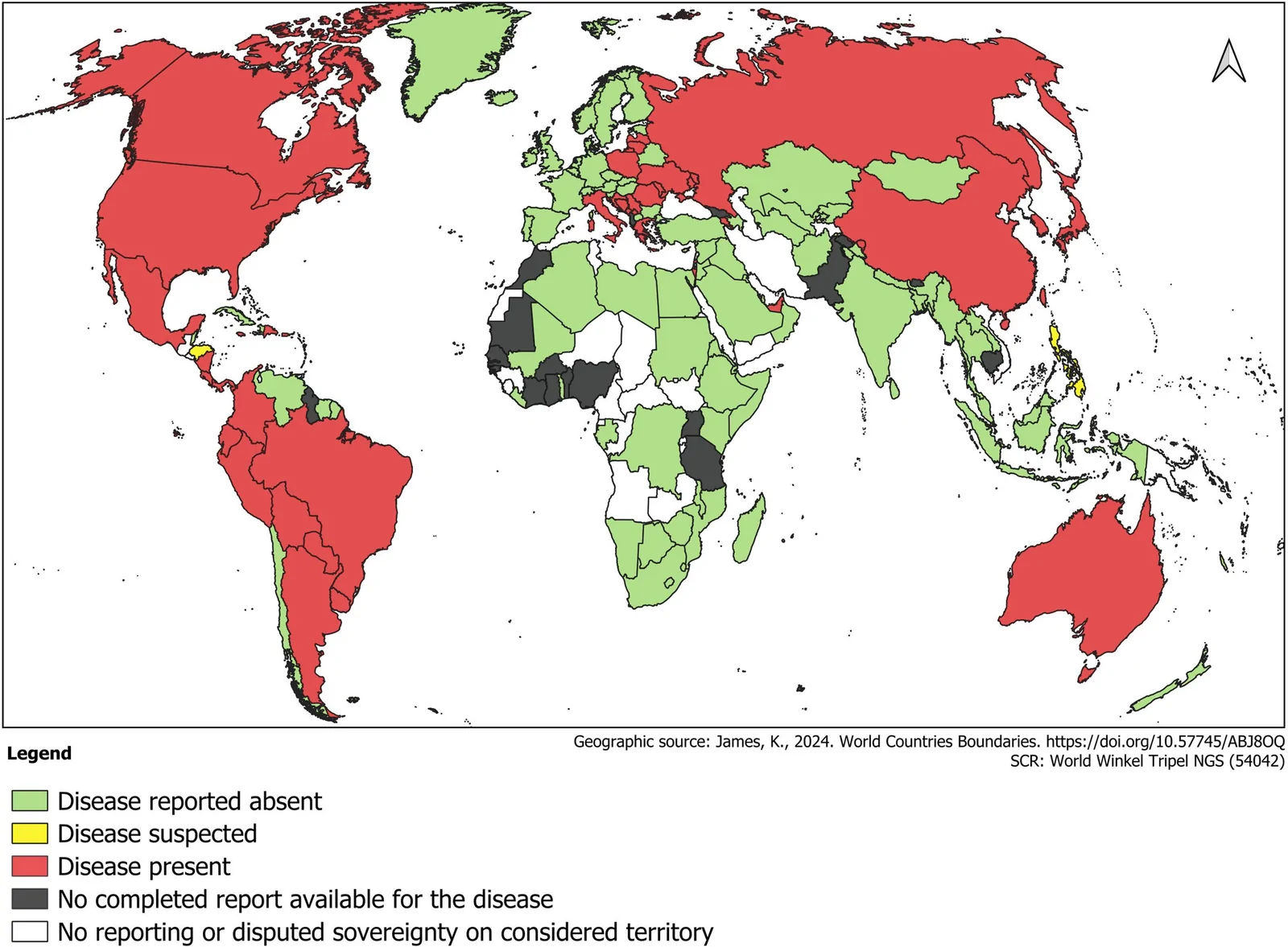
Enzootic bovine leukosis disease situation reported to the world animal health information system (WAHIS) by countries and territories for 2024

This global distribution (Fig. 2) shows the presence of the disease across the entire American continent, with large livestock-producing countries notably affected by BLV, such as Argentina, Brazil, Canada, and the US. BLV has also been recorded in Oceania and Asia, with cases reported in Australia, Japan, and Russia. Some Asian countries (India, Vietnam, Indonesia), as well as most Western European countries reported no cases of the disease in 2024, while most Eastern European countries still declared active infections. Finally, the African continent and the Middle East were characterized by a heterogeneous landscape of countries where the presence of BLV was either unknown or officially reported as absent. However, these patterns should be interpreted with caution, as the absence of reported cases does not necessarily indicate the absence of infection. Differences in surveillance intensity, diagnostic capacity, and reporting practices between countries may contribute to the observed geographic distribution.

Based on a compilation of data from the WOAH information system and the available literature mapped to their respective countries or regions, we propose the following interpretative classification of the global epidemiological situation of BLV into three groups of countries or territories:

- Countries without a national or supra-national control program, where ad hoc monitoring or serological surveys show both BLV herd-level prevalence and within-herd prevalence rising, especially in dairy herds. Such countries include, for example, the US (LaDronka et al. 2018), Japan (Murakami et al. 2013; Arai et al. 2026), Canada (Bongers et al. 2025), Korea (Suh GukHyun et al. 2003), and China (Zhao et al. 2024; Wang et al. 2026).
- Countries where control measures have successfully reduced BLV prevalence to very few cases, resulting in a disease-free status according to WOAH standards and EU regulations, e.g., France and Germany, or according to EU regulations yet nonetheless classified by WOAH as still affected (e.g., Poland and Italy). Similarly, Australia and New Zealand implemented national eradication programmes from the 1990 s onwards, resulting in a substantial reduction in BLV prevalence (Kirkland and Rodwell 2005; Ryan 2013). However, residual cases, mainly reported in Australian beef herds, explain why Australia continues to be classified by WOAH as an affected country (Queensland Government 2025).
- Countries reporting zero cases to WOAH, although this profile should be interpreted in light of the research and surveillance intensity. For example, BLV has been documented in several countries such as Kenya, Myanmar, or Egypt (Kiugu 2018; Hamada et al. 2020; Moe et al. 2020), while corresponding WOAH reports indicated no cases during the same period (WAHIS 2026b).

### European situation with respect to BLV

The first EBL control measures were implemented in Europe, even before its etiologic agent had been identified (EFSA AHAW Panel 2015). It followed the detection, at the end of the nineteenth century, of EBL as a contagious form of bovine lymphoma, and its identification as a disease causing direct economic losses due to carcass condemnation (Bendixen 1965a, 1973). Consequently, management programs were introduced in 1959 in Denmark that specifically targeted cattle with persistent lymphocytosis (Bendixen 1973). Within the European Union, early regulatory measures against EBL addressed both food safety—through veterinary post-mortem inspection—and active herd surveillance, notably via large-scale, exhaustive blood sampling campaigns. Animals with persistent lymphocytosis were culled, and hygienic measures were enforced alongside state-led financial compensation schemes (Council of the European Economic Community 1964; Council of the European Communities 1977). Over time, these regulations were progressively adopted by the first nine Member States of the European Economic Community (Belgium, Denmark, France, Germany, Ireland, Italy, Luxembourg, the Netherlands, and the United Kingdom), and eventually, extended to all new Member States.

**Fig. 3:**
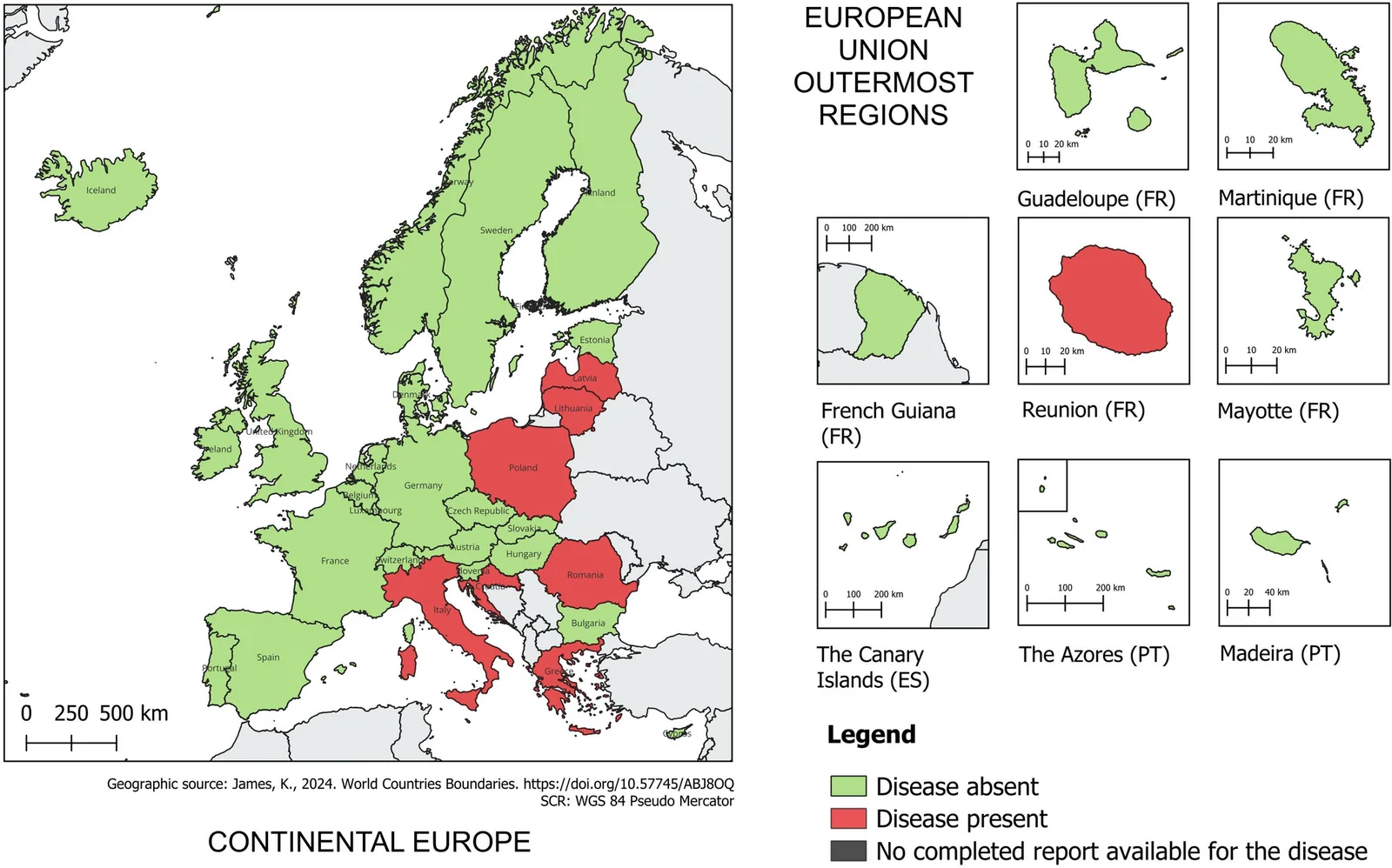
Enzootic bovine leukosis disease situation reported to the world animal health information system (WAHIS) by EU member States, their ORs, and partner countries for 2024

**Fig. 4:**
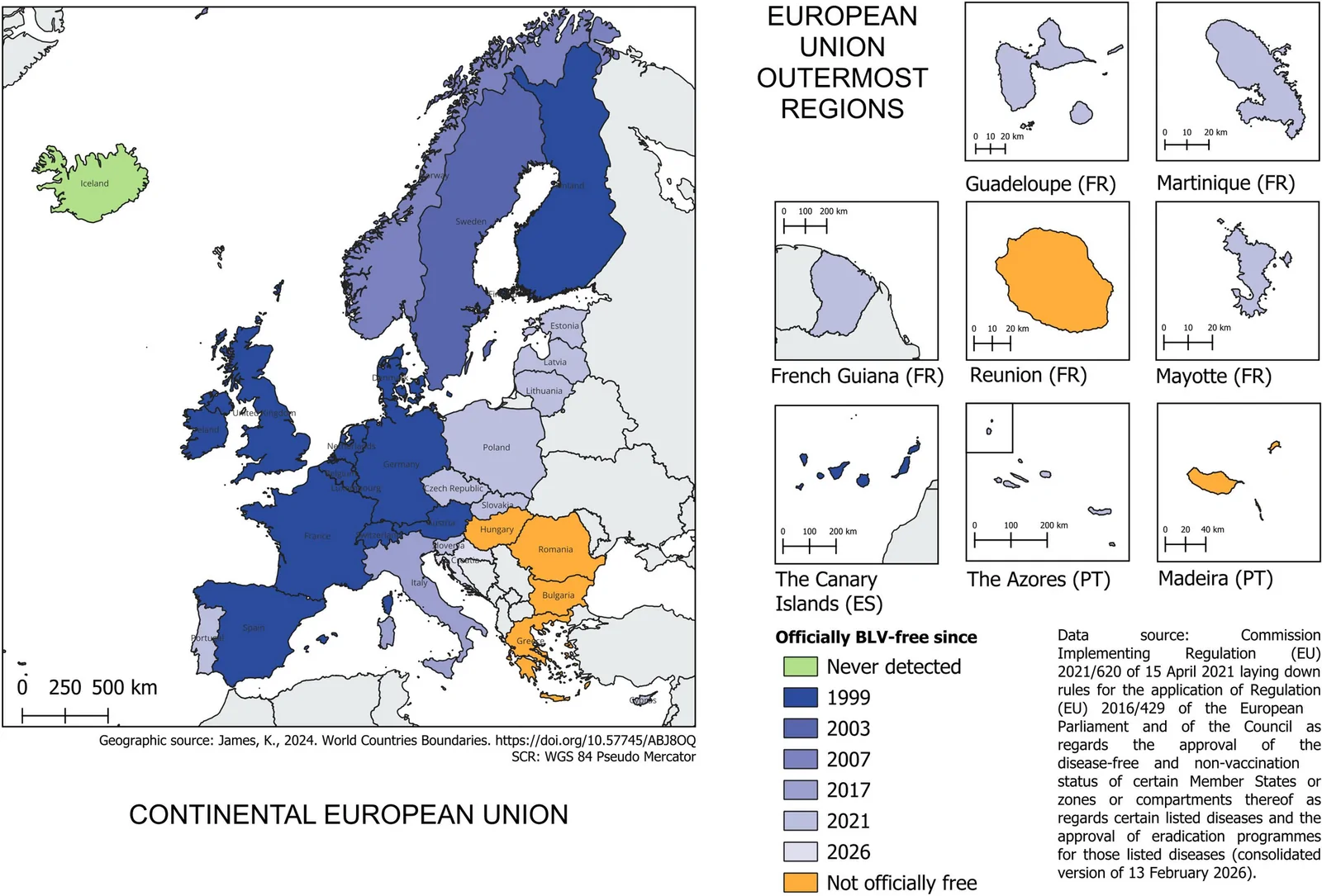
Official status map of enzootic bovine leukosis disease for European Union Member States, including their ORs, and partner countries as of EU Commission Implementing Regulation (EU) 2021/620 (last updated February 13, 2026)

The BLV status for each EU Member State was retrieved using data extracted from WOAH and EU regulations, all publicly available (Figs. 3 and 4, and Appendix I). All the countries in the European Economic Community, except Italy, were recognized as BLV-free in 1999, together with Austria, Finland, Spain (including its OR of the Canary Islands), and Switzerland (Commission of the European Communities 1999). Following the creation of the European Union, this status was obtained by Sweden and Norway in 2003 and 2007, respectively (Commission Of The European Communities 2003). After application of an eradication plan for more than 20 years, Italy was officially declared free from BLV in 2017 (European Commission 2017), despite the persistence of some infectious clusters (Righi et al. 2021). In 2021, seven Eastern European countries were recognized as BLV-free, including the Czech Republic, Estonia, Latvia, Lithuania, Poland, Slovakia, and Slovenia (European Parliament and European Council 2021). In the same year, two additional countries—Cyprus and Portugal (including one of its ORs, the Azores)—as well as most French ORs (French Guiana, Guadeloupe, Martinique, and Mayotte) acquired the BLV-free status. Croatia adopted a national eradication program which was approved by the EU in 2022, leading to the recognition of its BLV-free status in 2026 (European Commission 2026b). In 2024, a total of 28 European countries or territories were considered BLV-free according to WOAH regulations (WOAH 2018, 2024; WAHIS 2025), and by February 2026, 21 EU Member States had an EU-recognized BLV-free status (European Commission 2026a). Non BLV-free territories fall into two categories: 5 Member States, predominantly situated in Southeastern Europe (Bulgaria, Greece, Hungary, Malta, and Romania), and two outermost regions that are both island territories (Madeira/Portugal and Reunion/France). The French OR of Reunion Island has been implementing a local control plan since 2020.

**Fig. 5:**
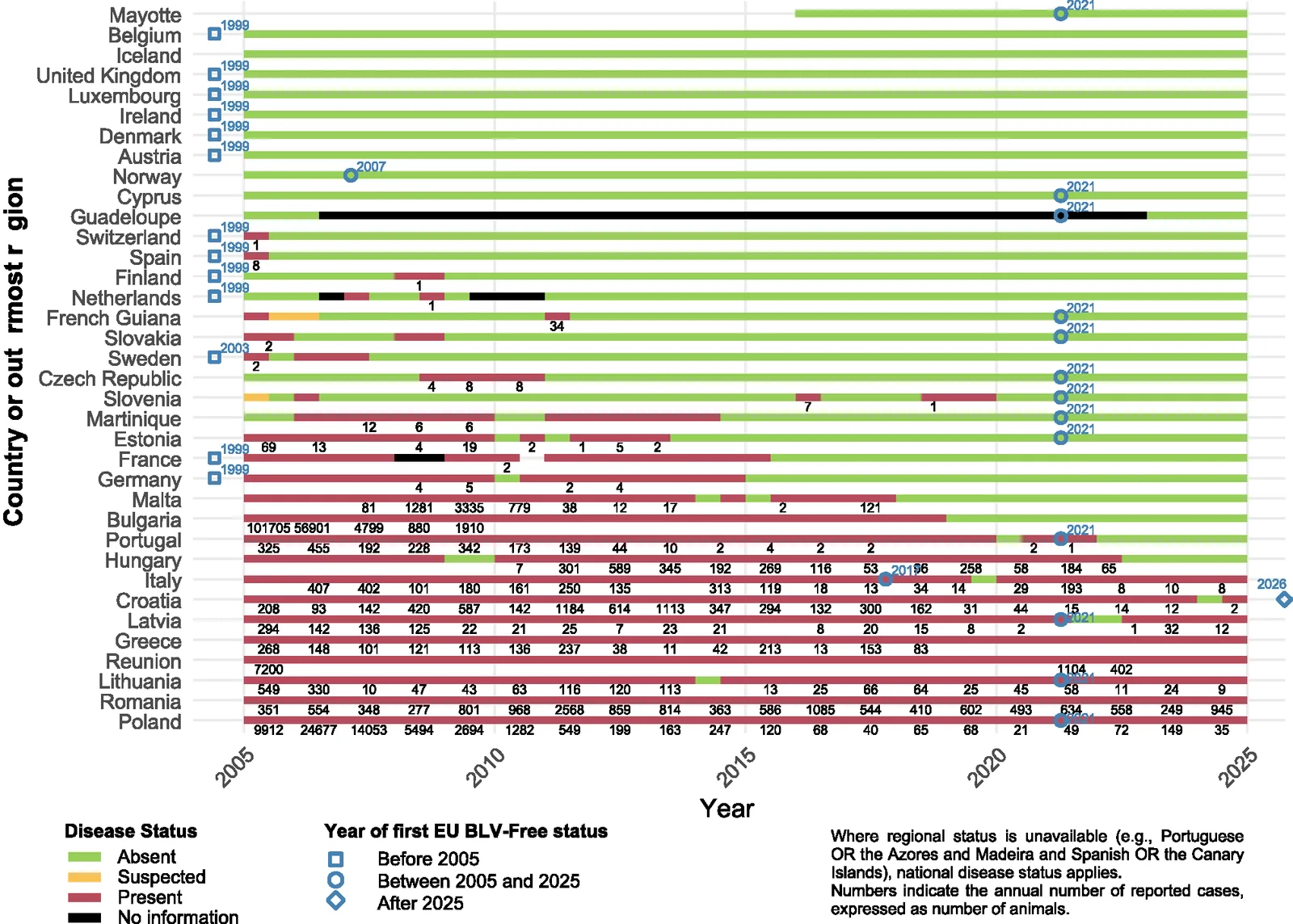
Enzootic bovine leukosis disease situation and number of cases reported to the world animal health information system (WAHIS) by EU member States and their ORs, and partner countries from 2005 to 2024 included

Quantitative eradication results obtained by EU members since 2005 were plotted using both official EU status, WOAH disease status, and number of cases reported since 2005 for EU Member States and their ORs, in addition to partner countries (Fig. 5). Notably, the number of BLV cases was much higher in Eastern Europe than in other parts of the continent, accounting for 98% of reported cases during the last 20 years, as seen through the case numbers e.g., in Latvia, Lithuania, Poland, and Bulgaria. Even several years after achieving the requirements set by EU legislation to acquire BLV-free status, sporadic cases of BLV still occurred in countries like France, Germany, Italy, Latvia, Lithuania, and Poland. The main challenge observed in the final stage of BLV eradication in most European countries appears to be the exponential decline in case numbers. Any country or region embarking on a BLV management plan may anticipate a “control plan tail” phenomenon at the end of the program, where the decline in the number of cases stagnates for several years before achieving total eradication. Additionally, other countries reported sporadic emergence during the few years following official approval of its eradication, as in Finland and the Netherlands. It is noteworthy that in both the stagnating and sporadic emergence situations, the number of reported cases nonetheless remained below the threshold required to invalidate the EU’s official BLV-free status. Finally, some countries remained infected, like Greece and Malta, or Reunion Island, a French OR. Although the epidemiological situation improved over time, it remained heterogenous over the entire European territory (EFSA AHAW Panel 2015; EFSA AHAW et al. 2017). Several factors may contribute to the persistence of BLV in the remaining non-free Member States and outermost regions. First, cattle production systems are highly heterogeneous across Europe, ranging from highly specialized commercial farms to small family-operated or semi-subsistence holdings, which may influence both the feasibility and cost-effectiveness of eradication measures (European Commission and Eurostat 2022, 2023). Second, surveillance and control programs were implemented progressively across Europe, often reflecting national priorities, accession timelines to the EU framework, and trade incentives. Resource availability may also have played a role, as EU financial support for enzootic bovine leukosis eradication was discontinued in the mid-1990s (Toussaint 2021), potentially increasing the economic burden borne by countries pursuing eradication at a later stage. Finally, ecological and logistical constraints may complicate disease control in specific regions, including wetlands and mountainous areas where access to farms may be more difficult (Maresca et al. 2015; Irimia et al. 2021). In some territories, such as outermost regions with limited live-animal trade, authorities may also have prioritized available resources toward other animal health challenges perceived as more pressing (ANSES 2015). Combined with relevant literature, this led us to propose a distinction of at least three different epidemiological contexts for BLV across EU Member States, territories and regions: (i) persistent clusters of infection despite eradication efforts for many years (Greece, Italy, Poland); (ii) a drastic increase in BLV prevalence similar to the North American situation (Reunion Island/France); and (iii) special regulations and natural risks restricting the control measures available and linked to more extensive farming systems, with mountain areas being harder to reach, e.g., traditional Alpine pastures, or swamps in the Romanian Danube delta (Caminiti et al. 2017; Irimia et al. 2021; Righi et al. 2021; Pluta et al. 2026).

The underlying mechanisms behind these different results could potentially be linked to the heterogeneity of production systems within the EU, the most fundamental distinction being between dairy and beef operations. Higher prevalence in dairy than in beef cattle has suggested that management practices such as housing practices, cattle breed, production type and management of introduced replacement animals are key factors in BLV transmission (Hopkins and DiGiacomo 1997; Kuczewski et al. 2021b; Tani et al. 2025). Among beef cattle, there are several different production systems in Europe, both for the origin of fattening calves and the way of breeding cow-calf herds (Wilczynski 2018). The spatial distribution of cattle breeding is heterogeneous, showing regionalization: most of the bovine populations are located in the North of Europe or in mountainous areas, and dairy cows in particular are mainly bred in close clusters, in specialized regions where milk collection for processing is rationalized (European Commission and Eurostat 2022, 2023, 2024a, 2024b). Historically, European management plans for EBL control often had to face great heterogeneity at regional levels and between bovine management systems (EFSA AHAW Panel 2015). Reports have revealed significant differences in BLV prevalence between and within regions in the primary investigations and control plans conducted in France in the 1970 s and 1980 s (Lazar et al. 1967; Crespeau et al. 1978; Sandev et al. 2006), as well as in more recent programs conducted in Romania (Irimia et al. 2021) and Italy (Righi et al. 2021). Additionally, bigger herd size and the intensification of cattle handling may be favorable to the spread of BLV, especially in modern dairy herds (Mammerickx et al. 1985; Kobayashi et al. 2010). Furthermore, European agricultural production systems are also marked by a wide variation in herd density (livestock units per hectare or LSU/ha) between countries where intensive breeding systems are dominant and density range from 1.6 to 3.4 LSU/ha (the Netherlands, Belgium, Denmark) and those with mainly extensive systems (Bulgaria, Latvia, Lithuania, all three with 0.2 LSU/ha, (European Commission and Eurostat 2023). Most European island territories (Malta, Cyprus, French OR of Reunion Island) have very high livestock densities (European Commission and Eurostat 2023). Concomitantly, the general trend in Europe is a decrease in the number of livestock farms associated with an increase in their size, both in terms of the number of livestock and that of cultivated areas. Indeed, the number of EU dairy herds have decreased in the last few years, whereas milk yield per cow has improved (European Commission and Eurostat 2022, 2023, 2024a, 2024b). As a result, specific control programs tailored to these highly productive but often highly infected herds are required. In addition, extensive farming systems in natural areas, such as traditional free roaming bovine management in the Danube Delta, Romania or in Gargano National Park in Foggia, Italy, and herd gathering in the mountain pastures of the Alps (Caminiti et al. 2017; Irimia et al. 2021; Righi et al. 2021), represent unfavorable environmental conditions for the surveillance of animal health, and also require specific approaches.

## Prospective approaches for BLV management

### The emerging interests in controlling BLV

#### Economic impact of BLV at farm level

At the farm level, an evaluation of the direct economic consequences of BLV infection may differ depending on the type of economic loss accounted for, the system involved (free market versus supply chain-managed system), the production type (intensive versus traditional, dairy versus beef), and regional particularities. Japan, Canada, and the US are the most frequently analyzed situations (Ott et al. 2003; Kuczewski et al. 2019; Nakada et al. 2022, 2023; Watanabe et al. 2024). The direct impact of BLV on agricultural activities was first reported by Danish veterinary authorities as early as the 1960s. Slaughterhouse data revealed a ten-fold higher incidence of bovine myocarditis in infected areas in Denmark (28.7 cases per 100,000 head of cattle versus 1.5 cases) (Bendixen 1973). Indeed, the main economic losses associated with BLV arise from carcass condemnations after slaughter, from the death of animals before reaching the slaughter stage—either directly from EBL, from intercurrent diseases facilitated by BLV infection, or due to euthanization. In high-prevalence areas, e.g., in Canada and the US, lymphosarcomas attributed to BLV are considered to be the leading cause of condemnation of whole bovine carcasses (White and Moore 2009; Market and Industry Services for Agriculture and Agri-Food of Government of Canada 2024). More recent quantification of BLV impacts on farms has focused on two different aspects: i) study of the pathogeny of BLV to investigate what causes its indirect impacts on the bovine organism, and ii) economic outcomes in dairy systems with high within-herd prevalence (Nakada et al. 2022, 2023; Watanabe et al. 2024; Bourassi et al. 2025). For dairy cattle in Japan, a global decrease in carcass weight has been observed in carcasses entering the food chain (i.e., without lymphoma lesions and therefore not subjected to whole-carcass condemnation), with up to 30.4 kg in carcass weight loss for cows with a high PVL (Nakada et al. 2022). Given the 73,650 dairy cows slaughtered in Hokkaido over the studied year, the authors estimated the economic loss due to carcass weight loss to be US $1,391,649. Globally, many authors have described higher death and premature culling rates in BLV-infected herds, leading to a negative impact on cow longevity in dairy herds (Bartlett et al. 2013; Nekouei et al. 2016; Benitez et al. 2022). In a US study, 3,849 BLV-positive cows monitored across 112 Michigan herds were found to have a higher likelihood of death or culling than their BLV-negative herd mates (hazard ratio of 1.23, after adjusting for age and lactation rank) (Bartlett et al. 2013). Beef herds were also impacted, with reduced longevity directly reducing the number of calves produced per cow's lifetime (Benitez et al. 2020). A significant decrease in milk yield was observed in BLV-infected cows, with an impact on both milk quantity and quality (i.e., fat yields, somatic cell counts) (Ott et al. 2003; Choi et al. 2026). The estimated annual loss in milk yield was measured at 336 kg and 1,204 kg for infected cows and cows that had persistent lymphocytosis, respectively (Da et al. 1993). BLV also has indirect impacts on host and herd health. It has been hypothesized that synergistic interactions between BLV and other pathogens can increase overall susceptibility to disease. Indeed, pathogenesis studies have shown that BLV infection impairs immune system function, primarily by cell proliferation and alteration of immune homeostasis, cytokine and antibody production, and apoptotic response (Frie and Coussens 2015; Frie et al. 2016). Cattle naturally infected with BLV exhibited impaired adaptive immune responses to common diseases like Bovine Viral Diarrhea (BVD) and *Neospora caninum* (Menin et al. 2025). At the individual level, BLV-infected cattle are less susceptible to spontaneous recovery from dermatophytosis and are more prone to subclinical mastitis (Trainin et al. 1996; Ambrósio et al. 2021). At the herd level, BLV prevalence was associated with higher incidence of mastitis, gastroenteritis and bronchitis (Emanuelson et al. 1992). BLV infection in non-replacement dairy calves (males) was associated with more respiratory abnormalities and fewer symptom-free observations during an 84-day prospective study (Broadfoot et al. 2025). Most studies found a link between these impacts and the progression of BLV infection in vivo, allowing stratification of economic losses using a categorization of the cow's health status according to PVL levels. A cohort study conducted on 1,034 Holstein cows from nine Japanese dairy herds found that the cows with a high PVL were more subject to subclinical mastitis than cows not infected with BLV (hazard ratio 2.61) (Nakada et al. 2023). The resulting annual economic loss for these high PVL cows was estimated to be 2.73 times higher than that for aleukemic infected cows ($418.59 versus $152.96, respectively) (Nakada et al. 2023) . The annual losses potentially attributable to BLV infection in a typical Michigan dairy herd were estimated to range between $50,000 and $125,000, assuming a herd size of 550 cows and a within-herd prevalence of 46% (Frommelt 2023).

#### Economic and behavioral barriers to BLV control

Considering these estimates, controlling BLV appears to represent an economic necessity for bovine herds, in both beef and dairy sectors. However, financial constraints are frequently cited as a major obstacle to BLV control efforts, as several mitigation measures are time-consuming and costly. At the farm level, the cost–benefit balance of control measures has been estimated to strongly depend on the system and type of production (Kuczewski et al. 2019). The cost of control measures for highly specialized dairy herds with high prevalence is expected to be very high (Centers for Epidemiology and Animal Health 2008). Difficulties were encountered in implementing BLV control programs as early as the ‘80 s (Acaite et al. 2007). Today, farmers’ reluctance to cull BLV-infected animals at their own cost is still identified as a key factor behind the lack of motivation towards BLV control among US farmers (LaDronka et al. 2018), and the reason why stakeholders are calling for compensation by the government in Japan (Nakada et al. 2023). Such situations illustrate that decisions regarding disease control are not always driven by the maximization of long-term economic returns. For example, farmers may be reluctant to cull apparently healthy infected animals despite the expected future benefits of reduced BLV transmission, or may postpone the adoption of costly control measures because of immediate financial constraints. It has therefore been proposed that such factors should be integrated in micro-economic modeling for animal health to complement epidemiological modeling and support both private and institutional policymakers when designing and evaluating interventions (Rat-Aspert and Krebs 2013). This approach draws on the concept of bounded rationality (Simon 1967), which suggests that farmers make “satisficing” rather than fully optimizing decisions, under conditions of imperfect information, cognitive constraints, and uncertainty (Quinet 1994). However, farmers’ behavior cannot be reduced to bounded rationality alone. Recent research in the social sciences has highlighted the importance of social norms, institutional contexts, trust, and risk perceptions in shaping decisions related to disease control. These findings suggest that analyses of farmers’ willingness to adopt control measures should also consider the broader social and institutional factors influencing decision-making, including the role of public authorities (Maye et al. 2023).

#### Implications for livestock value chains and trade

Costs associated with BLV can also be assessed through national (or other territorial unit) value chains of livestock production. At the sector level, BLV seropositivity in US herds has been associated with a loss of $285 million to producers (Ott et al. 2003). The costs borne by non-farm stakeholders often impact farmers indirectly, and can also be estimated. Indeed, the impact of disease may cause an economic imbalance between supply chain actors. In the US, estimates have suggested that BLV-related reductions in milk yield increased milk prices and decreased consumer surplus (Losinger 2006), leading to a yearly loss for consumers of $240 million (Ott et al. 2003). Like in the US, agriculture is a key sector in the EU: agricultural production contributed 1.2% to the EU's GDP, with more than 45% of the EU’s 9.1 million farms keeping livestock (European Commission and Eurostat 2023). In 2020, a survey indicated that almost half of EU livestock units were cattle (European Commission and Eurostat 2023). The European herd comprises 74 million cattle in 2023, including 20 million dairy cows producing an average of 7,800 kg of milk per cow per year (European Commission and Eurostat 2024b). Given the structural importance of livestock production within the European agricultural sector, similar value-chain effects could reasonably be expected in the EU, even though they have not yet been quantified for BLV.

#### Implications for veterinary public health

For public authorities, the relevance of BLV control extends beyond farm-level economic losses and includes the costs associated with surveillance and disease prevention programs, borne by either publicly funded veterinary services or privately owned veterinary companies and laboratories paid by farmers. Surveillance activities may involve testing live animals, monitoring animal movements, and meat inspection procedures, with their intensity depending on the epidemiological situation of a given country or region (Somura et al. 2014). In the European Union, long-term eradication programs have successfully reduced BLV prevalence to very low levels in most Member States, allowing many countries to achieve officially BLV-free status. Nevertheless, maintaining this favorable situation requires continued surveillance and regulatory measures to prevent re-emergence. Beyond surveillance costs, BLV status may also influence access to animal and germinal product markets, as current European and national regulations seek to limit the spread of the virus via live animal movements and germinal product trade (EFSA AHAW et al. 2017). Export restrictions have historically imposed substantial economic costs on cattle farms (Bürki 1978). This challenge is amplified in the EU, where up to 15% of the total EU bovine livestock may cross intra-community borders each year (Directorate-General for Health and Food Safety (European Commission) 2024). Consequently, officially BLV-free status provides important guarantees for trade, whereas EU members that have not achieved this status are barred from accessing high-value markets for breeding stock and germinal products, resulting in significant missed economic opportunities.

BLV is one of the bovine diseases that act as an immunosuppressive factor facilitating other infections. Through its effects on the immune system, BLV alters host responses to both pathogens and vaccines, leading to reduced antibody titers and abnormal immune cell responses in infected cattle compared with non-infected ones (Erskine et al. 2011; Puentes et al. 2016; Frie et al. 2016). As a result, BLV contributes to broader patterns of poor animal health. This has important implications beyond the farm level, including negative effects on the public image of livestock production and, more critically, interference with state-led disease control programs. Bovine tuberculosis (bTB) provides a clear illustration of this dynamic. bTB is a zoonotic bovine disease on the decline in the EU, although still present in many Member States, including those with long-standing eradication programs (e.g., France, Italy, and Germany) (Rankova and Balieva 2025). As a result, it is still subject to strict test-and-cull control policies based on intradermal tuberculin testing. BLV infection has been identified as a risk factor for bTB progression, increasing *Mycobacterium bovis* burden and disease severity, while also reducing skin-test sensitivity in infected animals (Menin et al. 2025). In regions where BLV circulates, these interactions may complicate bTB eradication efforts. Conversely, the question of whether BLV could occur due to iatrogenic transmission during bTB skin tests was evaluated in the 1980s. Routine bTB skin testing did not result in BLV transmission, but tests performed using blood-contaminated tuberculin needles led to BLV seroconversion (Roberts et al. 1981; Evermann et al. 1986). More broadly, the increasing reliance on testing and vaccination campaigns to manage emerging and re-emerging animal diseases may heighten the risk of iatrogenic BLV transmission, thereby reinforcing the role of BLV as a structural constraint on effective veterinary public health interventions. Recent outbreaks, such as lumpy skin disease in Western Europe, illustrate how rapid screening and mass vaccination campaigns are central to disease management. BLV-induced immunosuppression and altered vaccine responses in infected cattle may act as a cofactor facilitating disease spread in highly enzootic settings. Among these emerging diseases, the spread of highly pathogenic avian influenza (HPAI) since the 2010 s highlights the zoonotic potential of emerging pathogens (Runstadler and Puryear 2024). It also sparked interest in an HPAI vaccination campaign for US cattle herds, in an attempt to reduce the frequency of its cross-species transmission from poultry to cattle and humans. Because BLV has been found to be associated with immunosuppression and altered responses to vaccination in bovine organisms, it may act as a cofactor that weakens the effect of vaccination and facilitates the spread of HPAI in highly enzootic countries. Altogether, these findings suggest that BLV infection may diminish the accuracy of diagnostic methods and the protective immunity conferred by vaccination—two crucial components in veterinary public health programs.

#### Zoonotic hypotheses and societal concerns

BLV was identified as a potential zoonotic concern in the 1960 s as it belongs to the deltaretrovirus genus (International Committee on Taxonomy of Viruses 2025) and is closely related to the Human T-lymphotropic virus type 1 (HTLV-1) associated with lymphomas in humans (World Health Organization 2024). One frequently cited study at the time described the fatal erythroleukemia developed in two chimpanzees that were fed unpasteurized milk from naturally BLV-infected cows (McClure et al. 1974). As a result, the zoonotic potential of BLV was considered relevant when the first control policies were set up in the EU (Theilen et al. 1968). However, the available evidence remained inconclusive, and subsequent reviews concluded that BLV did not warrant public health interventions in cattle (Burridge 1981). More recently, Buehring and her colleagues detected anti-BLV antibodies in human blood sera and proviral sequences in US breast cancers (Buehring et al. 2003, 2014). Similar results were reported from South America (Argentina, Columbia, Brazil), Asia (Iran and Pakistan) and Australia (Nikbakht Broujeni et al. 2010; Buehring et al. 2017; Bartlett et al. 2020; Canova et al. 2021; de Quadros et al. 2023). Based on case control studies, BLV was found to be significantly more prevalent in breast cancers than in normal and benign breast tissue controls (Khatami et al. 2020; Lawson and Glenn 2024; Saeedi-Moghaddam et al. 2024). Conversely, other studies failed to detect BLV-related antibodies or sequences in human from different countries (US, China, Korea, Japan) and did not support any association between BLV and breast cancers (Lee et al. 2005; Zhang et al. 2016; Gillet and Willems 2016; Saito et al. 2020; Adekanmbi et al. 2021; Yamanaka et al. 2022; Amato et al. 2023). These conflicting results have been tentatively attributed to differences in the performance of the diagnostic methods (ELISA, immunohistochemistry, nested PCR, in situ PCR, whole genome sequencing), the variability of targeted viral sequences (mutations and partial genome deletions), sample sizes and study designs, and epidemiological differences between human cohorts in the previously reported studies, such as genetic predisposition, environmental exposures and dietary influences that would affect likelihood of BLV infection (Blanco et al. 2025). Overall, the association between BLV and human breast cancer remains highly controversial and unresolved. Nevertheless, scientists have called for further research, and recommend control programs in cattle anyway, even before scientific evidence has been obtained (Gyles 2016). In this context, potential concerns related to human health and consumer perceptions—such as fear-driven reductions in meat consumption—may arise. In at least one context, the subject received extensive media coverage and fueled consumer distrust: the French OR of Reunion Island was subject to a media crisis, followed by a boycott of bovine products by consumers. They were joined by political figures relaying demands for control measures in cattle entering the food chain under the precautionary principle (Malet 2017; Ramassamy 2018; Decloitre 2019). However, these dynamics have remained largely underexplored by social science research in the case of BLV, despite a growing literature addressing similar mechanisms in other animal health crises, such as bovine spongiform encephalopathy (BSE). Applying social science perspectives to BLV would help to elucidate these processes without reducing them to notions of collective hysteria but instead situating them within broader dynamics of risk perception, trust, and public controversy.

### Options for controlling BLV in 2026

#### Diagnostic tools

So far, various methods have been used to detect BLV infection, bearing in mind its specific behavior as an integrated provirus, and the persistence of the host’s humoral immune response for the lifetime of the infected animal. Blood analysis methods like lymphocyte counts are well described in the literature (Bendixen 1963, 1965b, 1973), but frequently give false negative results (Mammerickx et al. 1978), since only 30% of infected animals display changes in blood cell counts (Frie and Coussens 2015; WOAH 2018). Antibody detection using serological methods (namely agar gel immunodiffusion – AGID - and enzyme-linked immunosorbent assay - ELISA) is currently the recommended approach due to its good performance and moderate cost. These methods typically identify antibodies directed against the p24 capsid protein and the gp51 envelope glycoprotein. ELISA is a sensitive and cost-effective method suitable for high-throughput screening on both sera and milk samples, including pooled samples, while AGID is preferentially used as a confirmatory test. There are three situations where serological tests can produce inaccurate results (either false positive or false negative): i) recently infected animals which have not yet developed antiviral antibodies (3–4 weeks post-infection), ii) calves having received colostrum and milk from BLV-seropositive dams (detectable up to 6–7 months of age) (John et al. 2024), and iii) cattle in specific physiologic states (e.g., peripartum) (WOAH 2018). The direct detection of BLV through PCR is the only way to confirm (or refute) an infection in these serological blind spot situations. All three generations of PCR (conventional single or nested PCRs, real-time PCRs and digital PCRs) have been developed for BLV diagnosis. To date, real-time PCR tests are the most commonly used to detect BLV proviral DNA (Pluta et al. 2024). Although more expensive, these PCR-based methods provide a confirmatory test to complement serology (WOAH 2018). These tests can detect the infection several weeks before seroconversion, and can also distinguish between BLV-positive and non-infected calves in the presence of maternally-derived antibodies. However, PCRs on peripheral blood mononuclear cells (PBMC) may fail to detect some infected but asymptomatic animals due to a very low PVL in blood (fewer than two copies per 50 ng of genomic DNA in blood samples), the infection being hidden in lymphoid tissues rather than in circulating lymphocytes (WOAH 2018; Mekata et al. 2018). The advent of real-time quantitative PCR (qPCR) tests opened the way to estimating PVL in peripheral blood. A lower-cost alternative is the use of total white blood cell count (WBCC) as a marker of PVL. Although a strong correlation between WBCC and blood PVL has been reported (Alvarez et al. 2013), its use lacks sensitivity. Some cows with a normal WBCC may present a high PVL and can thus be a source of infection (Ruggiero et al. 2019). The limitations of qPCR methods are their high cost and their quantification accuracy: Results from two inter-laboratory comparisons of PCR assays for BLV detection demonstrated substantial discordance in PVL measurements (Jaworski et al. 2018; Pluta et al. 2024). Recently, digital PCR assays were developed to detect and quantify BLV in blood samples (De Brun et al. 2022; Notsu et al. 2023; Wu et al. 2023). This last-generation PCR appears highly specific, more sensitive than qPCR, and can determine the absolute copy number of BLV provirus with minimal PCR bias. Moreover, similar performances were achieved using unpurified genomic DNA (Wu et al. 2023), making digital PCR a cost-effective method suitable for large-scale screening.

#### Conventional control strategies

There are currently no curative treatments, effective vaccines, or preventive therapy against BLV (WOAH 2018; Bartlett et al. 2020). Therefore, the main recommendation to eradicate it in a given herd is to identify infected cattle through testing, followed by their culling or physical segregation, and to implement management practices designed to limit the spread of BLV within the herd (Bartlett et al. 2014, 2020; Kuczewski et al. 2019). Test-and-cull programs have been widely implemented in all European countries since the ‘60 s, first using hematological tests and then immunological detection (AGID and ELISA). They led to a very steep decline in both herd level and animal level prevalence (Roch and Conrady 2021; Irimia et al. 2021; Righi et al. 2025), especially when applied in low to medium prevalence regions (Crespeau et al. 1978; Mammerickx et al. 1985; Sandev et al. 2006). In high prevalence areas, management authorities may consider—like those in North America in 2007—that the test-and-cull strategy was not cost-effective (Centers for Epidemiology and Animal Health 2008). Test-and-segregate programs were identified as an alternative to culling. This approach has been evaluated only to a limited extent and under specific climatic conditions (temperate climate in Korea) (Suh et al. 2005). The effectiveness of such programs in different environmental conditions, especially with regard to the potential variability of BLV transmission under different housing or vector density, remains to be determined in larger studies.

The implementation of better hygienic measures was found to decrease within-herd prevalence, without achieving eradication (Kuczewski et al. 2021a). At the herd level, hygienic measures considered the most effective for limiting BLV spread and preventing its re-emergence in a herd are as follows (Juliarena et al. 2017):

1. Use of individual disposable needles and syringes for blood extractions and injections.
2. Use of individual obstetric gloves for transrectal palpation.
3. Use of disposable or adequately disinfected equipment for all procedures carrying a risk of blood contamination, such as dehorning, castration, ear-tagging, etc.
4. Feeding of calves with milk from BLV-negative cows, milk replacer, or heat-inactivated milk from BLV-positive cows. Colostrum can be frozen, which is considered sufficient to destroy the infectious cell walls while sparing the maternal antibodies that are essential for the calf. It can then be thawed and distributed.
5. Use of artificial insemination or natural breeding with BLV-negative bulls.
6. Introduction of BLV-negative animals from negative herds, or strict isolation of newly introduced animals until a negative test is obtained (e.g., two BLV-negative serological examinations performed at 2 to 4 months’ interval) if coming from infected herds or herds with an unknown status.
7. Control of blood-sucking insects. This measure is not widely supported, especially in North American studies, which have pointed out a lack of knowledge about the role of insect vectors in BLV transmission (Kuczewski et al. 2021b), whereas it has been recommended in both South American and Japanese studies (Igawa 2015; Juliarena et al. 2017).

#### BLV control measures implemented worldwide

**Fig. 6:**
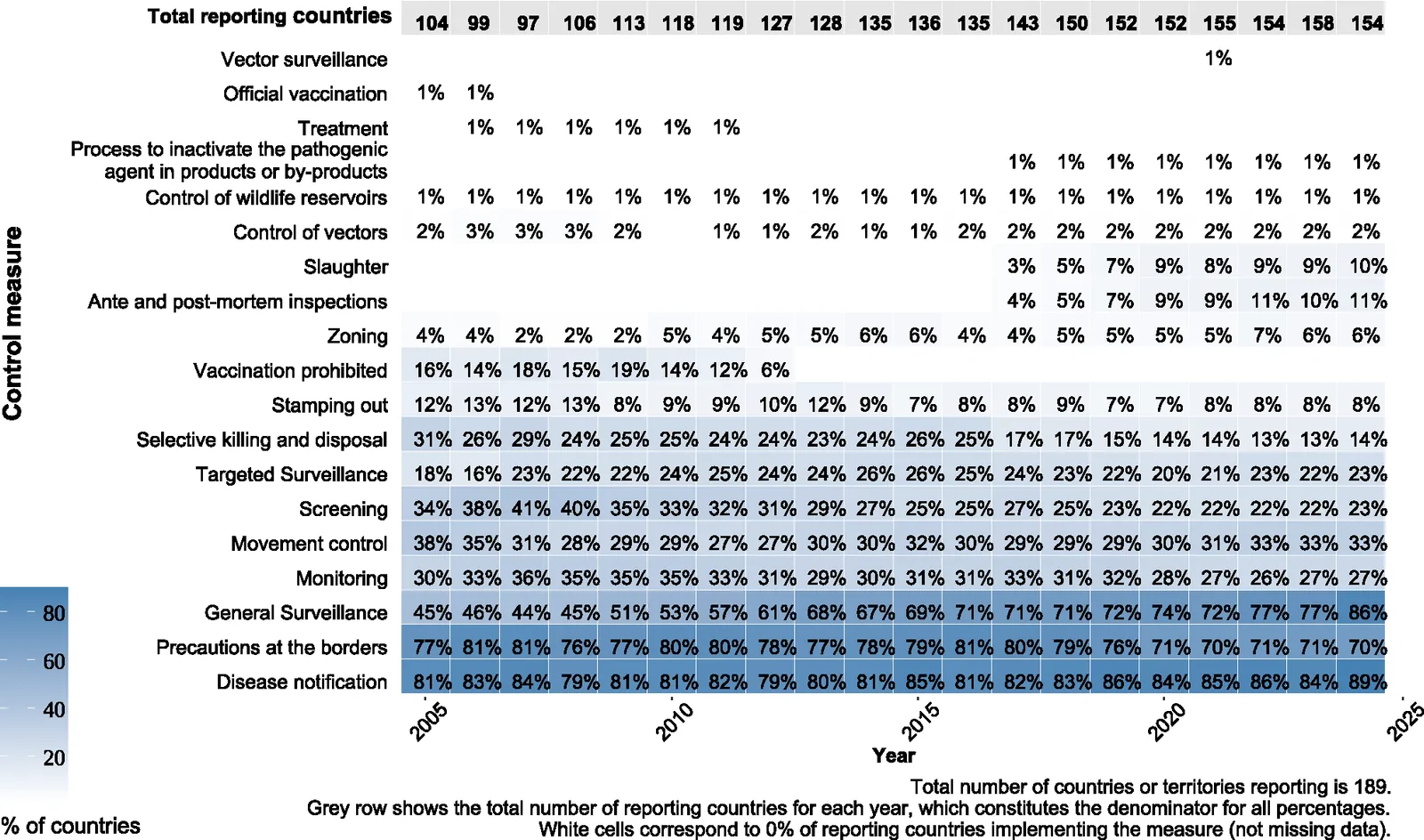
Annual percentage of countries implementing each BLV control measure reported to WAHIS from 2005 to 2024 included

At the state level, we analyzed BLV control plans from 187 WOAH members reporting to WAHIS (WAHIS 2026a) between 2005 and 2024 inclusive (Fig. 6 and Appendix IV). Control measures were organized by decreasing overall frequency of reporting across all the years. Nineteen different control measures were listed, the most popular being precautions at the borders, general surveillance, monitoring, and movement control (Appendix III). Sporadic reports of treatment or official vaccination—as well as prohibition of vaccination—appeared inappropriate, as neither tool has ever been commercially available. Three eradication procedures for outbreaks were reported, all using a test-and-cull approach; 1) stamping out, 2) selective killing and disposal, and 3) slaughter. According to the WOAH guidelines (WOAH 2025a), stamping out is defined as the killing of all affected, suspected and exposed animals, with destruction of carcasses and animal products, followed by cleaning and disinfection. Selective killing and disposal refer to the same measures but applied only to infected animals. Finally, slaughter refers to the killing for disease control purposes, with carcasses intended for commercial use (Appendix III). Selective killing and disposal was the most frequently reported eradication procedure in 2024 (14%, *n* = 21 members), followed by slaughter (10%, *n* = 15 members), then stamping out (8%, *n* = 12 members). For the past 20 years, there has been a trend towards postponement, with fewer members reporting selective killing and disposal or stamping out, and more favoring slaughter. At the same time, ante- and post-mortem inspections have become a widely reported measure, used by 11% of members (*n* = 17). In 2024, only three Member States reported the implementation of vector control measures.

#### The European regulatory framework

We then compiled the relevant EU regulations on BLV in order to highlight the measures that have been implemented and have resulted in a favorable status for most of Europe (Appendices V 5 and 6 VI). The EU legal framework is common to all members, and expected to be uniformly applied across all EU Member States. It aims to i) promote the eradication of BLV in countries or zones where it is present and ii) protect BLV-free countries or zones from re-infection (EFSA AHAW et al. 2017). As a result, it prohibits transboundary trade of cattle and their offspring from any herd that is not officially free of the disease, as well as prohibiting the integration of such animals into any production facility for germinal products (semen, oocytes and embryos intended for artificial reproduction) (European Parliament and Council of European Union 2016; European Commission 2023). Since April 21 st, 2021, a new EU regulation and its delegated acts (Directive 2016/429) on transmissible animal diseases, known as the “Animal Health Law”, have entered into force and are directly applicable to all EU Member States, binding all relevant stakeholders (European Parliament and Council of European Union 2016; European Commission 2018, 2019, 2023, 2026a; European Parliament and European Council 2021). These regulations classify BLV as a category C + D + E disease, making it a notifiable disease subject to optional eradication programs (cat C, see Appendix VI). This updated legal framework defines the conditions required to achieve and maintain BLV-free status at national or regional scales, thereby offering guarantees for intra-EU trading of live animals and importation of bovine semen and embryos (European Commission 2018, 2019, 2026a; European Parliament and European Council 2021). In 2021, EBL was the disease most frequently targeted by optional control programs across the EU (Costa et al. 2020; Hodnik et al. 2021).

Hence, official BLV-free status in the EU relies on precise specifications for its acquisition, maintenance, suspension, withdrawal, and recovery, both at the herd, or “establishment”, and country level (European Commission 2019). Under European Union regulations, an “establishment” refers to any premises, structure, or—under open‑air farming conditions—any environment or location where animals are kept on a temporary or permanent basis, excluding private households keeping pet animals and veterinary practices or clinics (European Parliament and Council of European Union 2016). An establishment can obtain BLV-free status if i) no confirmed case has occurred in the past two years, ii) adult cattle (> 24 months) have tested seronegative twice within the previous year, and iii) introduced animals and germinal products comply with strict origin and testing criteria. The status is then maintained through continued compliance with these requirements and periodic serological surveillance, typically conducted every three years, unless favorable conditions at the regional level allow a reduction in surveillance frequency. The surveillance is performed through periodic serological controls on a fraction of cattle over 24 months of age, typically using ELISAs. The establishment’s BLV-free status is suspended when maintenance requirements are not met or infection is suspected. Restoration then requires renewed compliance and evidence of the absence of the disease.

Withdrawal of an establishment’s BLV-free status follows persistent non-compliance or confirmation of BLV infection. Herds with unfavorable results are placed under restrictive measures, including a ban on animal movements (i.e., sale, transfer, boarding, or exhibition at shows) and the use of their germinal products. The BLV-free status may be regained if i) any bovines that tested positive and all of their offspring younger than 24 months of age are removed, and if ii) all remaining animals older than 12 months test negative twice within an interval of at least 4 months after a minimum of 4 months following the removal of positive animals. By derogation, offspring from animals that tested positive may be kept if they test negative by PCR, according to a dedicated testing protocol. At the regional level, acquisition of BLV-free status requires near-complete absence of BLV among herds (> 99.8% of establishments). Maintenance relies on structured monitoring through surveillance plans and systematic veterinary visual examinations at post-mortem meat inspection. During the first five years after being granted BLV-free status, the surveillance plan should be able to detect BLV-infected establishments at a target herd-level prevalence rate of 0.2% with a 95% level of confidence. After maintaining the officially free status for at least five consecutive years, national programs may allow a relaxation of surveillance measures, according to a risk-based sampling plan. Additionally, all lymphoid tumors detected in carcasses undergo histopathological analysis and non-negative tests trigger herd-level serological testing of all animals from the affected livestock holding.

#### Emerging and innovative control approaches

The marked increase in BLV prevalence has stimulated the development of alternatives to the traditional BLV control strategies in bovine herds. Several innovative tools to manage on-farm BLV risks have been developed and proved efficient at least through experimental studies since the 2000s. Integrating these tools into a BLV control strategy requires considering their associated costs and benefits, which are often only partially calculable. However, their integration can be readily addressed within sectoral policy discussions. We now present four innovative control methods, ranked by order of increasing effectiveness as demonstrated at the herd level and within the relevant epidemiological compartment: vector control, identification of contagious animals, genetic selection, and vaccination.

Vector-borne diseases may spread across borders, following either animal trade or passive expansion of arthropod populations. Within the EU, the ecological continuity between neighboring countries is likely to have promoted transboundary viral spread, as highlighted by epidemiological studies. Taking into consideration the European climate and natural conditions, the European Food Safety Agency (EFSA) considered unlikely that the vector-borne pathway could induce transmission between non-BLV-free and BLV-free areas, which is why there are currently no regulations regarding insect vector control (EFSA AHAW Panel 2015). However, at the farm level, vector control has proven effective in reducing BLV prevalence in Japanese herds (Kohara et al. 2006; Ooshiro et al. 2013). In 2024, three territories reported vector control as a BLV management measure to WOAH (WAHIS 2026a), namely France (French OR of Reunion Island) (Ministère de l’Agriculture et de l’Alimentation 2020), Japan (Igawa 2015), and Syria (WAHIS 2026a). However, these measures require substantial financial and human resources. Notably, no single control measure applied alone has ever yielded satisfactory results in controlling stable flies (*Stomoxys* spp.). In the European context, effective control has only been achieved through integrated strategies combining biological control agents (either predatory wasps or mites), continuous trapping of adults, and hygienic measures including the removal of bedstraw (where larvae grow) (González et al. 2024). Additionally, sterile insect techniques are considered a promising option for stable fly control, although they remain costly and have not yet been deployed at an operational scale (Bouyer and Marois 2018).

In farms with high BLV prevalence, approaches based on systematic testing and culling or segregation of all infected animals are considered economically unsustainable (Centers for Epidemiology and Animal Health 2008). As a result, a “test and cull of highly contagious cows” approach has been developed. Infectivity tests conducted by North American and Asian research teams confirmed that cows with a low PVL are generally not contagious under normal farming conditions (Mekata et al. 2018), while cows with a high PVL have been found more often on farms with high within-herd prevalence (Fujii et al. 2025; Arai et al. 2026). These findings led to the categorization of infected cattle into two PVL profiles or phenotypes: animals with a low or undetectable PVL are recognized as BLV controllers and non-transmitters, while animals with a high PVL are at higher risk of being a source of transmission (threshold level at 10,000 provirus copies/10^5^ cells in blood) (Juliarena et al. 2016; Ruggiero et al. 2019). As a result, the selective removal of animals with a high PVL can lead to a drastic decrease in the within-herd prevalence and incidence of new BLV infections (Ruggiero et al. 2019; Bartlett et al. 2020; Shrestha 2024; Bourassi et al. 2026). Screening seropositive cattle by quantitative PCR and subsequently segregating or culling animals with the highest PVLs is an alternative approach relevant to BLV control in herds with high prevalence, especially when relying on self-replacement of heifers (Gutiérrez et al. 2014; Hutchinson et al. 2020, 2021).

In addition to PVL, genetic polymorphism in the bovine lymphocyte antigen system (BoLA), particularly exon 2 of the DRB3 gene, has also received focus as a marker of BLV transmission and disease susceptibility. A few DRB3 alleles were found to be associated both with PVL and clinical outcome, which allowed their classification as resistant or susceptible alleles (International Society for Animal Genetics 2025). In naturally BLV-infected cattle, the stronger association between DRB3 variants and a low PVL was allele 009:02. Experimentally-infected calves carrying this resistant allele displayed a PVL that progressively declined to zero (Forletti et al. 2020). A three-year follow-up study confirmed that naturally-infected cows carrying this allele maintained undetectable BLV provirus levels in milk (Nakatsuchi et al. 2022). Introducing infected cows having a 009:02 heterozygous status and a low PVL into a BLV-free dairy herd at an average ratio of 5.4 BLV-negative cows per BLV-infected cow during a contact period of 20 months did not lead to new infections (Juliarena et al. 2016). A recent study on four Japanese dairy farms showed that they were able to effectively prevent new infections over three years by combining usage of this resistant allele and selective culling. They used BLV-positive cows carrying the resistant 009:02 allele as a biological barrier between BLV-negative cattle and infected cattle, and also culled infected cows with a high PVL or susceptible alleles (Borjigin et al. 2023). Altogether, these findings indicate that genotyping DRB3 alleles could be an additional tool for controlling BLV spread. This approach is constrained by the limited number of alleles with known phenotypes, the low frequency of resistant alleles in cattle populations, and substantial PVL variability among cattle carrying the same resistant/susceptible alleles. Indeed, zygosity at the DRB3 gene is associated with variation in PVL (Daous et al. 2021; Lo et al. 2021), where heterozygous cattle tend to exhibit a lower PVL, likely due to broader antigen recognition and the dominance of protective alleles. Further research is currently needed to identify allelic combinations that confer resistance/susceptibility to BLV infection and spreading. Additional work could evaluate the resources needed to implement such a control method relying on genetic selection targeting BLV resistance at the herd level, and on a broader scale of the value chain.

To date, there are currently no safe and effective commercial vaccines available to control BLV infection in cattle (WOAH 2018). Vaccines against retroviruses are challenging to develop because these viruses induce a lifelong persistent infection through stable integration of their genetic material into the host genome, thereby escaping immune response. Many previous attempts to develop a BLV vaccine (inactivated viruses, crude cell lysates, viral subunits, synthetic peptides, recombinant vaccine virus, DNA vectors) failed to provide complete protection against infection (Abdala et al. 2019). More recently, cumulative results from reverse genetic approaches led to the establishment of a live attenuated BLV candidate with promising vaccine potency (Suárez Archilla et al. 2022). A vaccine trial in a dairy herd with a high BLV incidence revealed that 28 out of 29 (96.5%) naive calves inoculated with a single dose of this vaccine strain developed a long-lasting antibody response, and remained protected from infection during the observed period (48 months), even when housed with unvaccinated, BLV-infected herd mates, and gave birth to calves with undetectable proviral loads during the first two production cycles. The vaccine strain was genetically stable, replicated at extremely low levels, and could only be detected sporadically and at low levels in the milk of vaccinated cows, rendering unlikely both the reversion to the pathogenicity of the parental strain and its transmission to offspring and susceptible herd mates. However, sterilizing immunity was not assessed, and concerns about insertional mutagenesis persist. Consequently, large-scale trials using a wild-type virus to challenge vaccinated animals are still needed to confirm both efficacy and biosafety. Moreover, beyond the technical and safety issues raised by scientists and authorities, the future availability of an effective BLV vaccine would not automatically guarantee its widespread adoption. As discussed above, farmers’ disease-control decisions are influenced by economic considerations, as well as broader social, organizational, and psychological factors like risk perception and trust in institutions (Maye et al. 2023). Consequently, the practical impact of vaccination will depend not only on vaccine efficacy and biosafety, but also on its acceptability among stakeholders.

Overall, BLV control currently relies on a combination of established and emerging approaches (Table 2). Test-and-cull, animal segregation, and hygienic measures remain the cornerstone of control programs, whereas vector control and PVL-driven strategies may provide valuable complements in specific epidemiological contexts. Genetic selection and vaccination represent promising longer-term options but still require further validation before large-scale implementation.

**Table 2:** Overview of established and emerging BLV control options and their current level of validation for field implementation

Control option	Principle	Evidence level	Effective use in the field	Main limitations
Test-and-cull	Removal of infected animals	Strong field evidence	Proven eradication strategy; widely implemented in Europe and historically successful in low- to moderate-prevalence settings	Expensive in moderate- to high-prevalence herds; may become economically unsustainable
Test-and-segregate	Separation of infected and non-infected cattle	Limited but supportive field evidence	Alternative to culling in high-prevalence herds; successfully implemented under specific management conditions	Logistically demanding; requires dedicated facilities, labor, and long-term compliance
Hygienic measures	Reduction of iatrogenic and horizontal transmission	Good supporting evidence	Routine component of BLV control programs and herd-level biosecurity	Generally insufficient alone to achieve eradication
Vector control	Reduction of insect-mediated transmission	Context-dependent evidence	Relevant mainly in tropical or subtropical settings (e.g., Reunion Island, Japan); reported by only a few territories	Costly, labor-intensive, and effectiveness depends on local ecology
PVL-driven control	Identification and removal of highly infectious animals	Growing field evidence	Particularly useful in high-prevalence herds where whole-herd test-and-cull is impractical	Requires quantitative PCR testing and repeated monitoring
Genetic selection	Selection of cattle carrying resistant BoLA alleles	Promising experimental and field evidence	Successfully tested in a limited number of dairy herds when combined with selective culling	Limited knowledge of resistance-associated alleles; low frequency of favorable genotypes
Vaccination	Induction of protective immunity	Experimental evidence only	No field deployment; candidate vaccine showed promising results under experimental conditions	No commercial vaccine available; efficacy and biosafety require further validation

### How social analysis could ensure better control plans

BLV-associated risks, costs, and opportunities are usually assessed within the bounds of the agricultural economy. They can also be assessed while including social and political aspects, with a whole-of-society approach. Within the agricultural sector, when a control strategy is decided upon, the main factor dictating its efficiency is the quality of its implementation. The engagement of farmers in the strategy is often analyzed through the lens of the acceptability of measures, which is supposed to depend on individual knowledge and attitude toward disease control tools. However, while knowledge and information matter, deficits in technical understanding alone do not explain acceptance or resistance to animal health policies (Jenkins and Gomez 2026). Social science research underlines that a good understanding of the engagement of farmers should include analysis of governance arrangements, which are the structured sets of institutions, actors, rules, and interaction patterns that organize decision-making, coordination, and implementation in a given policy domain. Its definition integrates all actors involved in policy-making and service delivery, building on governance theory that sees governing as distributed, networked, and often non-hierarchical rather than purely state-centered. Its analysis includes farmers’ trust in institutions and other professional groups, as well as perceived policy fairness; that is, whether constraints are seen as equitably distributed, rules consistently applied, and compliance fairly assessed (Gates et al. 2021). Tools to assess these governance arrangements include stakeholder mapping, sociology of professions such as veterinarians (Berdah 2021; Bonnaud and Fortané 2021), sociology of interest groups, and policy analysis (Porter 2012; Chien 2013; Figuié 2021) possibly including a historical dimension (Ollivier 2013). Although these issues have been extensively studied in relation to antimicrobial resistance or endemic diseases such as bovine tuberculosis, they have, to our knowledge, rarely been examined in the case of BLV.

Acceptability for stakeholders can also be assessed beyond the agricultural sector. Comparison with other diseases over time, drawing on past crisis management experiences, helps to identify what triggers the spillover of the animal health issue into the broader society as a wider political crisis. Comparative implementation studies—such as those conducted on tuberculosis, foot and mouth disease, or the 1990 s BSE crisis—make it possible to identify side effects, bargaining dynamics and gaps between expected and actual policy impacts (Millstone and van Zwanenberg 2001; Donaldson et al. 2002; Olmstead and Rhode 2004, 2007). Consequently, control programs targeting animal diseases may prove substantially more expensive than initially anticipated by the authorities in charge. A current illustration is the ongoing protest movement against the eradication campaign for lumpy skin disease in France, which has become a focal point for broader agricultural grievances, notably those linked to the European Union’s free trade agreement with Mercosur. BLV has also been associated with at least one instance in which such hidden burdens transformed into political penalties for local authorities and caused an erosion of institutional legitimacy. Although this example should not be considered representative of all BLV control programs, it illustrates how animal health issues may become politically contested under specific local conditions. In the French OR of Reunion Island, the topic of BLV management received extensive media coverage and fueled criticism of state-led policies, ultimately politicizing and contesting what was previously only a technical issue (Malet 2017; Ramassamy 2018; Decloitre 2019). This case reflects a particular regional context, yet it provides an informative example of how local social, political, and institutional dynamics may shape the implementation and public perception of disease-control programs. This comparative perspective is particularly valuable in the current context of deeper shifts in the public and institutional representation of the agricultural sector, as well as changes in how states relate to farmers as a distinct social group (Mc Loughlin 2024), which fundamentally reshape the conditions under which animal health policies are implemented. Accordingly, the success of BLV control programs could be improved by addressing them within a broader framework that accounts for the socio-historical construction of veterinary public health issues, the social network structures shaping policy framing, and the need to anticipate potential “political costs”, meaning governance challenges, stakeholder resistance, and losses of public trust associated with animal health management programs.

## Conclusion

Epidemiological analyses have provided a global overview of the distribution of BLV and the outcomes achieved through existing control efforts worldwide. BLV management mainly relies on veterinary and zootechnical tools, whose effectiveness has been demonstrated through their large-scale implementation across Europe. In parallel, a growing body of recent research has identified new technical approaches that may further strengthen BLV control, particularly in high-prevalence herd settings. These developments include identification of highly infectious animals, advances in genetic selection for resistance, and the emergence of vaccination tools. While these options require additional research to assess their full potential—especially in terms of expected reduction in prevalence and mitigation of economic impacts at the herd level—they are expected to progressively expand the range of options available to farmers and veterinarians, even under the challenging epidemiological conditions associated with a global increase in BLV prevalence. Recent economic assessments have also improved characterization of the BLV burden at both herd and value-chain levels. However, substantial uncertainties remain regarding the magnitude of these costs, as well as the propensity of individual farmers to integrate such estimates into their decision-making processes. In addition, difficulties persist in incorporating climate change projections into BLV management strategies, as robust estimates of the role of arthropod vectors under varying climatic and farm management conditions are still lacking, and the extent to which climate change may amplify the impacts of BLV remains unknown at this time.

These technical uncertainties are compounded by challenges in accounting for the social costs associated with both the disease and its state-led management, dimensions that remain largely underexplored despite their central importance in public eradication strategies targeting infectious animal diseases more broadly. Indeed, the social and political costs of BLV control—and of the absence of control—remain insufficiently investigated in terms of its implications for governance challenges, stakeholder resistance, and losses of public trust associated with animal health management programs. However, there is growing interest in these research topics related to infectious bovine disease control programs in general, and to BLV control programs in particular (Kuczewski et al. 2019). Indeed, the modern definition of veterinary public health is linking animal and human health, welfare, ecosystems and sustainable development within a One Health perspective (WOAH 2022; Food and Agriculture Organization of the United Nations et al. 2023). From a preventive veterinary medicine perspective, improving BLV control therefore requires not only effective technical measures but also greater attention to social costs. These costs may be significant, as some farming communities have developed increasingly critical attitudes toward state institutions and regulatory interventions, making the implementation of purely top-down policies more challenging (Mc Loughlin 2024). Since authorities are tasked with ensuring the acceptance of VPH programs and anticipating implementation difficulties, technical aspects extensively developed in reviews targeting BLV need to be supplemented by strategic and social considerations. This perspective is in agreement with findings that animal health operates as a multi‑level public good, shaped by governance arrangements and cross‑sectoral interactions rather than by technical views alone (Scoones and Forster 2008; Figuié 2013, 2021). Previous experience in animal health governance suggests that such moves do not dilute epidemiological objectives; instead, they stabilize cooperation, reduce unplanned political costs, and may accelerate progress towards freedom from BLV (Berdah 2021; Boschiroli 2023). Overall, integrating social cost considerations into the control of infectious animal diseases supports the implementation of dedicated policies through sustained participation, and enhances the long‑term effectiveness of VPH programs. Social costs could be explicitly integrated into the design and evaluation of BLV control programs. These costs arise at multiple levels—ranging from time and financial burdens to coordination difficulties and political effects such as contestation or loss of legitimacy—and are often difficult to quantify. Systems-thinking approaches grounded in a One Health perspective offer a useful framework for addressing these challenges by examining how surveillance instruments, stakeholder engagement, and feedback mechanisms interact (Lambraki et al. 2021). Empirical and comparative research adopting this perspective may strengthen BLV control strategies, foster more nuanced models of preventive VPH, and help anticipate both implementation constraints and opportunities associated with control programs.

Overall, the literature on BLV and its management highlights a series of interrelated constraints, requiring the integration of knowledge across multiple sectors and disciplines, including those that have been traditionally developed independently. Addressing such a disease therefore requires an integrated and unifying analytical framework capable of balancing and sustainably optimizing human, animal, social, economic, and environmental health. This perspective aligns with the One Health approach, which recognizes the interdependence of these dimensions and seeks to improve health outcomes through coordinated, cross-sectoral reasoning (WOAH 2022; Food and Agriculture Organization of the United Nations et al. 2023). Importantly, applying a One Health lens to this context implies moving beyond narrow economic calculations to include social—and political—costs as integral components of controlling infectious diseases. As demonstrated throughout this review, the relevance of such an approach is over-reaching, going from the direct impacts of the disease at the herd level to downstream economic effects along the value chain, and ultimately to the broader consequences of its integration into public policy. Approaches integrating social, economic, and organizational dimensions are progressing in the study of animal diseases with pronounced zoonotic or economic impacts. Although EBL is generally perceived as less critical in these aspects, many of the implementation challenges it raises closely mirror those documented for more frequently scrutinized diseases. Taken together, our contribution is less focused on adding a new disease-specific diagnosis than on demonstrating how a fully operationalized One Health approach can support more informed and balanced decision-making in veterinary public health when addressing infectious bovine diseases.

## Competing interests

The authors declare that they have no competing interests.

## Supplementary Information

Below is the link to the electronic supplementary material.

Supplementary file1 (DOCX 110 KB)

## Data Availability

The datasets analyzed during the current study are available in the WOAH repository, WAHIS database, at https://wahis.woah.org.

## Abbreviations

AGID: Agar gel immunodiffusion assay
BLV: Bovine leukemia virus
BoLA: Bovine lymphocyte antigen system
BSE: Bovine spongiform encephalopathy
EBL: Enzootic bovine leukosis
EFSA: European Food Safety Agency
ELISA: Enzyme-linked immunosorbent assay
EU: European Union
OR: Outermost region
PBMC: Peripheral blood mononuclear cells
PVL: Proviral load
qPCR: Real-time quantitative PCR
US: United States of America
VPH: Veterinary Public Health
WBCC: White blood cell count
WOAH: World Organisation for Animal Health
