# Supplementary material for: Integrating epidemiological, regulatory and social perspectives to improve bovine leukemia virus control
Source: Vet Res Commun. 2026 Oct 31;51(1):27. doi: 10.1007/s11259-026-11563-z (PMC13723676; doi:10.1007/s11259-026-11563-z)
Supplement: Supplementary file 1 [file 11259_2026_11563_MOESM1_ESM.docx]

Appendix I: Official status of enzootic bovine leukosis disease in European Union (EU) Member States, including their outermost regions, and partner countries as of EU Commission Implementing Regulation 2021/620 (consolidated version of 13 February 2026)

| Country/OR^a^ | EU entry | BLV status^b^ | Year^c^ |
| --- | --- | --- | --- |
| **EU member** |  |  |  |
| Austria | 1995 | Free | 1999 |
| Belgium | 1957 | Free | 1999 |
| Bulgaria | 2007 | Not officially free |  |
| Croatia | 2013 | Free | 2026 |
| Cyprus | 2004 | Free | 2021 |
| Czech Republic | 2004 | Free | 2021 |
| Denmark | 1973 | Free | 1999 |
| Estonia | 2004 | Free | 2021 |
| Finland | 1995 | Free | 1999 |
| France | 1957 | Free | 1999 |
| *French Guiana | 1957 | Free | 2021 |
| *Guadeloupe | 1957 | Free | 2021 |
| *Martinique | 1957 | Free | 2021 |
| *Mayotte | 1957 | Free | 2021 |
| *Reunion Island | 1957 | Not officially free |  |
| Germany | 1957 | Free | 1999 |
| Greece | 1981 | Not officially free |  |
| Hungary | 2004 | Not officially free |  |
| Ireland | 1973 | Free | 1999 |
| Italy | 1957 | Free | 2017 |
| Latvia | 2004 | Free | 2021 |
| Lithuania | 2004 | Free | 2021 |
| Luxembourg | 1957 | Free | 1999 |
| Malta | 2004 | Not officially free |  |
| Netherlands | 1957 | Free | 1999 |
| Poland | 2004 | Free | 2021 |
| Portugal | 1986 | Free | 2021 |
| *Azores | 1986 | Free | 2021 |
| *Madeira | 1986 | Not officially free |  |
| Romania | 2007 | Not officially free |  |
| Slovakia | 2004 | Free | 2021 |
| Slovenia | 2004 | Free | 2021 |
| Spain | 1986 | Free | 1999 |
| *Canary Islands | 1986 | Free | 1999 |
| Sweden | 1995 | Free | 2003 |
|  |  |  |  |
| **EU partner country** |  |  |  |
| Iceland |  | Free | NA^d^ |
| Norway |  | Free | 2007 |
| Switzerland |  | Free | 1999 |
| United Kingdom |  | Free | 1999 |
| ^a^ Outermost region  ^b^ Last updated 13/02/2026  ^c^ Year that the EU first recognized a BLV disease-free status  ^d^ Data not available | | | |

Appendix II: List of WOAH occurrence codes reported in the World Animal Health Information System

| Occurrence code | Meaning |
| --- | --- |
| Disease present | The disease is present with clinical signs in the whole country (in domestic species or wildlife). |
| Disease limited to one or more zones | The disease is present with clinical signs and limited to one or more zones/compartments (in domestic species or wildlife). To support the limited distribution of disease, it is important that evidence is provided through control measures such as disease notification, general surveillance, movement control, zoning and/or compartmentalization. |
| Infection/infestation | Infection* or infestation** confirmed by diagnostic testing in the absence of clinical signs (in domestic species or wildlife). |
| Infection/infestation limited to one or more zones | Infection or infestation confirmed by diagnostic testing in the absence of clinical signs and is limited to one or more zones or compartments (in domestic species or wildlife). To support the limited distribution of disease, it is important that evidence is provided through control measures such as disease notification, general surveillance, movement control, zoning and/or compartmentalization. |
| Disease absent | The disease was absent in the country during the reporting period (in domestic species or wildlife). This occurrence code should be used when there is confidence that the control measures in your national legislation would result in the detection and reporting of disease, if present. Control measures for assuring stakeholders of disease absence can be, but are not limited to, ‘disease notification’ and ‘general surveillance’. These should be in place for relevant susceptible domestic species, and for wildlife where wildlife is considered to be epidemiologically significant in accordance with the case definition in the Terrestrial Code. Disease absence cannot be declared if there are ongoing or stable events (in the WAHIS Early Warning Module) or if disease, infection or infestation is suspected. A date of last occurrence (date when the disease, infection or infestation was last declared absent by the Veterinary Authority) must be provided when the code is used. If this date is not known, then ‘Unknown date’ should be selected. To facilitate your reporting in WAHIS, the date of last occurrence is prefilled with the end date of the last outbreak of the most recent resolved event, or is transferred from the previous submitted report if the disease remained absent. In all cases where you need to edit the date of last occurrence and the full date is unknown, please select the last day of the known month or year (e.g., if 2010 is the only information available, the date to be entered is ‘2010/12/31’. Please provide an explanatory note directly in WAHIS if the prefilled date is modified. |
| Disease suspected | The presence of the disease was suspected but not confirmed in the whole country (in domestic species or wildlife). |
| Disease suspected and limited to one or more zones | The presence of the disease was suspected but not confirmed and limited to one or more zone or compartments (in domestic species or wildlife). |
| No information | No information is available regarding the presence or the absence of this disease during the reporting period (in domestic species or wildlife). Please note that for the purpose of disease reporting via WAHIS, ‘No information’ means that the relevant control measures in place (if any) are not able to provide enough confidence that the disease is present or absent in your country/territory during the reporting period. |
| Never reported | The disease has never been reported (historically absent) for the whole country in both domestic species and wildlife. The same requirements for ‘disease absent’ apply to this code. However, once a disease has been reported in the country with any code other than ‘never reported’, this code can no longer be applied to either domestic species or wildlife. |
| *Infection means the entry and development or multiplication of a pathogenic agent in the body of humans or animals. **Infestation means the external invasion or colonization of animals or their immediate surroundings by arthropods, which may cause clinical signs or are potential vectors of pathogenic agents. | |

Appendix III: List of WOAH control measures reported in the World Animal Health Information System

| Control measures | Meaning |
| --- | --- |
| Ante- and post-mortem inspections | Primary component of meat hygiene before slaughter and primary component of process control in post-slaughter meat hygiene. |
| Compartmentalization | Procedure implemented by a Member under the provisions of Chapter 4.4. and Chapter 4.5. of the Terrestrial Code to define an animal subpopulation contained in one or more establishments, separated from other susceptible populations by a common biosecurity management system, and with a specific animal health status within its territory for the purposes of international trade or disease prevention and control. |
| Control of vectors | Implementing measures to control insects or any living carrier that transports an infectious agent from an infected individual to a susceptible individual, its food or its immediate surroundings. |
| Control of wildlife reservoirs | Measures to reduce the potential for wildlife to transmit the disease to domestic animals and human beings (control of wildlife reservoir populations, vaccination of target wildlife, etc.). |
| Disease notification | Legislative framework is in place to support the reporting of any suspected or confirmed case of a disease, infection or infestation to the relevant authorities. |
| General surveillance | Surveillance not focused on any particular diseases, but rather capable of detecting any disease or pathogen. |
| Monitoring | Intermittent performance and analysis of routine measurements and observations, aimed at detecting changes in the environment or health status of a population. |
| Movement control | Measures aimed at avoiding the spread of the disease, infection or infestation within a country/zone/compartment due to the movement of animals or their products. |
| Official vaccination | Vaccination program, excluding emergency vaccination in response to the outbreak(s), which is approved and supervised by the Veterinary Authority. You can provide the number of animals vaccinated OR the vaccination coverage. These values are mutually exclusive, which means that you can only notify the number of animals vaccinated OR the vaccination coverage. The vaccine type used on a given species: if more than one vaccine type was used, please choose in WAHIS the vaccine type which was used most extensively in the official vaccination campaign. Important: • Please do not confuse this control measure with emergency vaccination in response to the outbreak(s), which is only done to control the spread of the disease and is available only in the WAHIS Early Warning Module. The vaccination coverage rate is the estimated percentage of individuals vaccinated in the target population of a given animal group. For example, in a population of 17,000,000 animals, the vaccination coverage for a given disease through official vaccination of 3,500,000 animals is 20.58% (3,500,000 / 17,000,000 x 100). If a value is entered for this field, no value can be entered for the field ‘Number of animals vaccinated’ and vice versa. |
| Precautions at the borders | Measures applied at airports, ports, railway stations or road checkpoints open to international movement of animals, animal products and other related commodities, where import inspections are performed to prevent introduction of the disease, infection or infestation into a country/territory or zone. |
| Process to inactivate the pathogenic agent in products or by-products | A set of management, chemical and physical measures designed to reduce the biochemical or biological activity of a substance or organism. |
| Screening | Testing carried out within the framework of a control program for the disease, infection or infestation, or for health qualification of herds/flocks in all or part of the national territory. Screening is usually disease-specific and should not be confused with general surveillance. However, the two control measures can coexist for the same disease. |
| Selective killing and disposal | Application of the measures described for ‘stamping out’ only on a group of animals within the susceptible population (e.g., killing and disposal of cases only). |
| Slaughter | Killing of animals for disease control purposes and intended for commercial use or own use. |
| Stamping out | Operation designed to eliminate an outbreak, carried out under the control of the Veterinary Authority, and consisting of the following activities: • The killing of the animals which are affected and those suspected of being affected in the herd or flock and, where appropriate, those in other herds or flocks which have been exposed to infection by direct animal to animal contact, or by indirect contact with the causal pathogenic agent. Animals should be killed in accordance with Chapter 7.6. of the Terrestrial Code. • The disposal of the carcasses and where relevant, animal products, by rendering, burning or burial, or by any other method described in Chapter 4.13 of the Terrestrial Code. The cleaning and disinfection of establishments through procedures defined in Chapter 4.14 of the Terrestrial Code. |
| Targeted surveillance | Surveillance focused on a specific disease or pathogen within a defined animal population. You can provide the number of animals sampled. If the number of animals sampled is not available, then the number of samples collected (more than one sample may be taken from the same animal) could be provided. |
| Treatment | Use of veterinary drugs for the purpose of controlling and curing the infection or infestation. This measure does not include supportive treatment. |
| Vaccination prohibited | As a general control policy, the use of a vaccine to control or prevent the disease or infection is prohibited. |
| Vector surveillance | Demonstrate the presence or absence of vectors to determine areas of different levels of risk, as well as local details of seasonality. It can be used to identify the various vector species present in an area, their respective seasonal occurrence, and abundance. |
| Zoning | Delineation defined by the Veterinary Authority, of part of a country/territory containing an animal population or subpopulation with a specific animal health status with respect to a disease, infection or infestation for which required surveillance, control, and biosecurity measures have been applied for the purposes of international trade or disease prevention and control, under the provisions of Chapter 4.4. of the Terrestrial Code. |

Appendix IV: Annual number of countries implementing each enzootic bovine leukosis control measure reported to the World Animal Health Information System from 2005 to 2024 inclusive

| **Indicator** | **Number of countries reporting the measure** | | | | | | | | | | | | | | | | | | | |
| --- | --- | --- | --- | --- | --- | --- | --- | --- | --- | --- | --- | --- | --- | --- | --- | --- | --- | --- | --- | --- |
| **Control measure** | **2005** | **2006** | **2007** | **2008** | **2009** | **2010** | **2011** | **2012** | **2013** | **2014** | **2015** | **2016** | **2017** | **2018** | **2019** | **2020** | **2021** | **2022** | **2023** | **2024** |
| Control of vectors | 2 | 3 | 3 | 3 | 2 | 0 | 1 | 1 | 2 | 1 | 2 | 3 | 3 | 3 | 3 | 3 | 3 | 3 | 3 | 3 |
| Control of wildlife reservoirs | 1 | 1 | 1 | 1 | 1 | 1 | 1 | 1 | 1 | 1 | 1 | 1 | 1 | 1 | 1 | 1 | 1 | 1 | 2 | 2 |
| Disease notification | 84 | 82 | 81 | 84 | 92 | 96 | 97 | 100 | 103 | 109 | 116 | 109 | 117 | 125 | 130 | 127 | 132 | 132 | 133 | 137 |
| General surveillance | 47 | 46 | 43 | 48 | 58 | 62 | 68 | 77 | 87 | 90 | 94 | 96 | 101 | 107 | 109 | 113 | 112 | 118 | 121 | 133 |
| Monitoring | 31 | 33 | 35 | 37 | 40 | 41 | 39 | 39 | 37 | 41 | 42 | 42 | 47 | 46 | 48 | 43 | 42 | 40 | 43 | 42 |
| Movement control | 39 | 35 | 30 | 30 | 33 | 34 | 32 | 34 | 39 | 40 | 43 | 40 | 42 | 43 | 44 | 45 | 48 | 51 | 52 | 51 |
| Official vaccination | 1 | 1 | 0 | 0 | 0 | 0 | 0 | 0 | 0 | 0 | 0 | 0 | 0 | 0 | 0 | 0 | 0 | 0 | 0 | 0 |
| Precautions at the borders | 80 | 80 | 79 | 81 | 87 | 94 | 95 | 99 | 99 | 105 | 107 | 109 | 115 | 118 | 116 | 108 | 108 | 109 | 112 | 108 |
| Screening | 35 | 38 | 40 | 42 | 40 | 39 | 38 | 39 | 37 | 37 | 34 | 34 | 38 | 37 | 35 | 34 | 34 | 34 | 35 | 36 |
| Selective killing and disposal | 32 | 26 | 28 | 25 | 28 | 30 | 28 | 30 | 30 | 32 | 36 | 34 | 24 | 25 | 23 | 22 | 21 | 20 | 20 | 21 |
| Stamping out | 13 | 13 | 12 | 14 | 9 | 11 | 11 | 13 | 15 | 12 | 10 | 11 | 11 | 13 | 11 | 11 | 12 | 12 | 12 | 12 |
| Targeted surveillance | 19 | 16 | 22 | 23 | 25 | 28 | 30 | 31 | 31 | 35 | 35 | 34 | 35 | 35 | 34 | 30 | 33 | 36 | 34 | 36 |
| Vaccination prohibited | 17 | 14 | 17 | 16 | 21 | 16 | 14 | 7 | 0 | 0 | 0 | 0 | 0 | 0 | 0 | 0 | 0 | 0 | 0 | 0 |
| Zoning | 4 | 4 | 2 | 2 | 2 | 6 | 5 | 6 | 7 | 8 | 8 | 6 | 6 | 7 | 8 | 7 | 8 | 11 | 10 | 10 |
| Treatment | 0 | 1 | 1 | 1 | 1 | 1 | 1 | 0 | 0 | 0 | 0 | 0 | 0 | 0 | 0 | 0 | 0 | 0 | 0 | 0 |
| Ante- and post-mortem inspections | 0 | 0 | 0 | 0 | 0 | 0 | 0 | 0 | 0 | 0 | 0 | 0 | 6 | 8 | 10 | 13 | 14 | 17 | 16 | 17 |
| Process to inactivate the pathogenic agent in products or by-products | 0 | 0 | 0 | 0 | 0 | 0 | 0 | 0 | 0 | 0 | 0 | 0 | 1 | 1 | 1 | 1 | 1 | 1 | 1 | 1 |
| Slaughter | 0 | 0 | 0 | 0 | 0 | 0 | 0 | 0 | 0 | 0 | 0 | 0 | 5 | 8 | 10 | 13 | 13 | 14 | 15 | 15 |
| Vector surveillance | 0 | 0 | 0 | 0 | 0 | 0 | 0 | 0 | 0 | 0 | 0 | 0 | 0 | 0 | 0 | 0 | 1 | 0 | 0 | 0 |
| **Total number of countries reporting** | **104** | **99** | **97** | **106** | **113** | **0** | **119** | **127** | **128** | **135** | **136** | **135** | **143** | **150** | **152** | **152** | **155** | **154** | **158** | **154** |

Appendix V: List of relevant European Union regulations

- European Commission, **2026**. Commission Implementing Regulation (EU) 2026/293 of February 9, 2026 amending Annexes I to IV, VII, VIII, XI and XIII and correcting Annex VIII to Implementing Regulation (EU) 2021/620 as regards the approval or withdrawal of the disease-free status of certain Member States, zones or compartments thereof as regards certain listed diseases and the approval of eradication programs for certain listed diseases (Text with EEA relevance). Off J Eur Union L 2026/293.
- European Commission, **2023**. Commission Delegated Regulation (EU) 2020/686 of December 17, 2019 supplementing Regulation (EU) 2016/429 of the European Parliament and of the Council as regards the approval of germinal product establishments and the traceability and animal health requirements for movements within the Union of germinal products of certain kept terrestrial animals (Text with EEA relevance). Off J Eur Union L174:1-63.
- European Commission, **2021**. Commission Implementing Regulation (EU) 2021/620 of April 15, 2021 laying down rules for the application of Regulation (EU) 2016/429 of the European Parliament and of the Council as regards the approval of the disease-free and non-vaccination status of certain Member States or zones or compartments thereof as regards certain listed diseases and the approval of eradication programs for those listed diseases (Text with EEA relevance). Off J Eur Union L131:78-119.
- European Commission, **2019**. Commission Delegated Regulation (EU) 2020/689 of December 17, 2019 supplementing Regulation (EU) 2016/429 of the European Parliament and of the Council as regards rules for surveillance, eradication programs, and disease-free status for certain listed and emerging diseases (Text with EEA relevance). Off J Eur Union L308:21-29.
- European Commission, **2018**. Commission Implementing Regulation (EU) 2018/1882 of December 3, 2018 on the application of certain disease prevention and control rules to categories of listed diseases and establishing a list of species and groups of species posing a considerable risk for the spread of those listed diseases (Text with EEA relevance.). Off J Eur Union L308:21-29.
- European Commission, **2017**. Commission Implementing Decision (EU) 2017/1910 of October 17, 2017 amending Decision 93/52/EEC as regards the brucellosis (B. melitensis)-free status of certain regions of Spain, Decision 2003/467/EC as regards the official bovine brucellosis-free status of Cyprus and of certain regions of Spain, and as regards the official enzootic-bovine-leucosis-free status of Italy, and Decision 2005/779/EC as regards the swine vesicular disease-free status of the region of Campania of Italy (notified under document C(2017) 6891) (Text with EEA relevance). Off J Eur Union L269:46-52.
- European Parliament, Council of European Union, **2016**. Regulation (EU) 2016/429 of the European Parliament and of the Council of 9 March 2016 on transmissible animal diseases and amending and repealing certain acts in the area of animal health (‘Animal Health Law’) (Text with EEA relevance). Off J Eur Union L84:1-208.
- Commission Of The European Communities, **2003**. 2003/467/EC: Commission Decision of June 23, 2003 establishing the official tuberculosis, brucellosis, and enzootic bovine leukosis-free status of certain Member States and regions of Member States as regards bovine herds (Text with EEA relevance) (notified under document number C(2003) 1925). Off J Eur Union L156:74-78.
- Commission Of The European Communities, **1999**. 1999/465/EC: Commission Decision of 13 July 1999 establishing the officially enzootic bovine leukosis-free status of bovine herds of certain Member States or regions of Member States (notified under document number C(1999) 2083) (Text with EEA relevance). Off J Eur Communities L181:32-33.
- Council of the European Communities, **1977**. Council Directive 77/391/EEC of May 17, 1977 introducing Community measures for the eradication of brucellosis, tuberculosis, and leucosis in cattle. Off J Eur Communities L145:44-51.
- Council of the European Economic Community, **1964**. Council Directive 64/432/EEC of 26 June 1964 on animal health problems affecting intra-Community trade in bovine animals and swine. Off J Eur Communities 121:1977-2012.

Appendix VI: Animal disease categories of the European Union Animal Health Law on transmissible animal diseases [Regulation (EU) 2016/429 of April 21, 2021]

| Disease | Definition |
| --- | --- |
| Category A | Listed disease that does not normally occur in the Union and for which immediate eradication measures must be taken as soon as it is detected, as referred to in Article 9(1)(a) of Regulation (EU) 2016/429 |
| Category B | Listed disease which must be controlled in all Member States with the goal of eradicating it throughout the Union, as referred to in Article 9(1)(b) of Regulation (EU) 2016/429 |
| Category C | Listed disease which is of relevance to some Member States and for which measures are needed to prevent it from spreading to parts of the Union that are officially disease-free or that have eradication programs for the listed disease concerned, as referred to in Article 9(1)(c) of Regulation (EU) 2016/429 |
| Category D | Listed disease for which measures are needed to prevent it from spreading on account of its entry into the Union or movements between Member States, as referred to in Article 9(1)(d) of Regulation (EU) 2016/429 |
| Category E | Listed disease for which there is a need for surveillance within the Union, as referred to in Article 9(1)(e) of Regulation (EU) 2016/429 |
